# Investigating the origins of eastern Polynesians using genome-wide data from the Leeward Society Isles

**DOI:** 10.1038/s41598-018-20026-8

**Published:** 2018-01-29

**Authors:** Georgi Hudjashov, Phillip Endicott, Helen Post, Nano Nagle, Simon Y. W. Ho, Daniel J. Lawson, Maere Reidla, Monika Karmin, Siiri Rootsi, Ene Metspalu, Lauri Saag, Richard Villems, Murray P. Cox, R. John Mitchell, Ralph L. Garcia-Bertrand, Mait Metspalu, Rene J. Herrera

**Affiliations:** 1 Statistics and Bioinformatics Group, Institute of Fundamental Sciences, Massey University, Palmerston North, Manawatu 4442 New Zealand; 2 Estonian Biocentre, Tartu, Tartumaa 51010 Estonia; 3 Department Hommes Natures Societies, Musée de l’Homme, 75016 Paris, Ile de France France; 4 Department of Biochemistry and Genetics, La Trobe University, Melbourne, Victoria VIC 3086 Australia; 5 School of Life and Environmental Sciences, University of Sydney, Sydney, New South Wales NSW 2006 Australia; 6 Integrative Epidemiology Unit, School of Social and Community Medicine, University of Bristol, Bristol, BS8 2BN United Kingdom; 7 Department of Molecular Biology, Colorado College, Colorado Springs, Colorado, 80903 USA

## Abstract

The debate concerning the origin of the Polynesian speaking peoples has been recently reinvigorated by genetic evidence for secondary migrations to western Polynesia from the New Guinea region during the 2nd millennium BP. Using genome-wide autosomal data from the Leeward Society Islands, the ancient cultural hub of eastern Polynesia, we find that the inhabitants’ genomes also demonstrate evidence of this episode of admixture, dating to 1,700–1,200 BP. This supports a late settlement chronology for eastern Polynesia, commencing ~1,000 BP, after the internal differentiation of Polynesian society. More than 70% of the autosomal ancestry of Leeward Society Islanders derives from Island Southeast Asia with the lowland populations of the Philippines as the single largest potential source. These long-distance migrants into Polynesia experienced additional admixture with northern Melanesians prior to the secondary migrations of the 2nd millennium BP. Moreover, the genetic diversity of mtDNA and Y chromosome lineages in the Leeward Society Islands is consistent with linguistic evidence for settlement of eastern Polynesia proceeding from the central northern Polynesian outliers in the Solomon Islands. These results stress the complex demographic history of the Leeward Society Islands and challenge phylogenetic models of cultural evolution predicated on eastern Polynesia being settled from Samoa.

## Introduction

**Figure 1:**
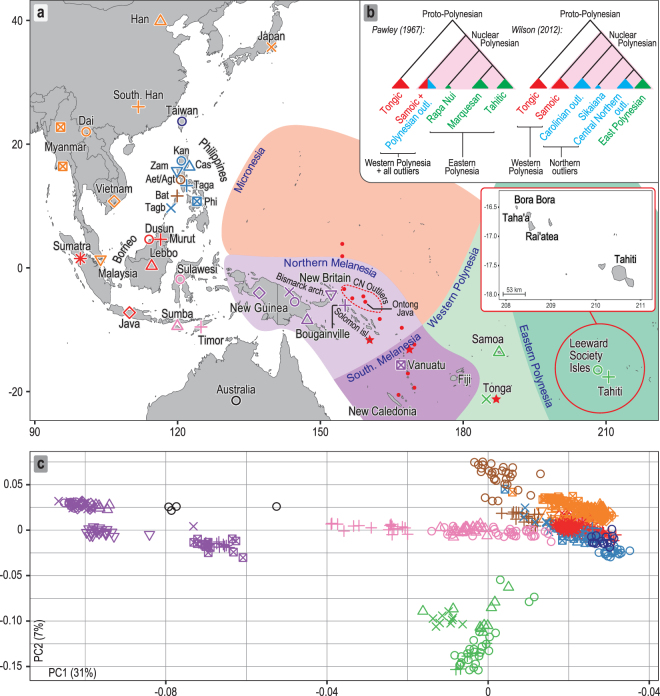
Sampling locations and overview of genomic diversity. (**a**) Sources of population data used in the present study. The Philippine group names are abbreviated as follows: Aet (Aeta); Agt (Agta); Bat (Batak); Cas (Casiguran); Kan (Kankanaey); Taga (Tagalog); Tagb (Tagbanua); Zam (Zambales); and Phi (Philippines, incorporating all other groups from this region). Colours indicate regional affiliation of populations used for analysis of autosomal DNA: orange – mainland Southeast Asia and East Asia; dark blue – Taiwan; brown – Philippines Aeta, Agta and Batak negritos; light blue – Philippines non-negritos; red – western Indonesia; pink – eastern Indonesia; purple – northern Melanesia and New Guinea; black – Australia; green –Polynesia. The usage of populations varies with the type of analysis employed (Supplementary Table S1). Inset map shows the three populations from the Leeward Society Isles, and Tahiti, the major island in the Windward Society Isles. The red circles within Micronesia and Melanesia represent 20 of the atolls and islands referred to collectively as outlier Polynesia. The red stars denote the three additional Polynesian outlier populations (Rennell and Bellona, Tikopia), which together with Tonga, were used in analysis of ancient admixture by Skoglund, *et al*.^25^. Detailed sample information is given in Supplementary Table S1. The map was created using R v. 3.4.1 (R Core Team (2017). R: A language and environment for statistical computing. R Foundation for Statistical Computing, https://www.R-project.org/), and packages ‘maps’ v. 3.2.0 (https://cran.r-project.org/package=maps) and ‘mapdata’ v. 2.2-6 (https://cran.r-project.org/package=mapdata). (**b**) Inset at top right shows two alternative reconstructed sub-groupings of Polynesian languages discussed in the text. The critical differences are the position of the East Polynesian languages relative to the rest of nuclear Polynesian, and their relationship to the Central Northern Outlier languages. In the sub-grouping according to Pawley^31^ all the Polynesian Outlier languages group within Samoic implying an early separation of Proto-East Polynesian from the rest of the Nuclear Polynesian languages. In the alternative sub-grouping proposed by Wilson^32^ the Central Northern Outlier languages group with the languages of East Polynesia, within a larger clade containing the other Northern Outlier languages. (**c**) Principal components analysis of genome-wide SNP diversity in 639 individuals populations shown in panel A; axes are scaled by the proportion of variance described by the corresponding principal component.

The cultural and linguistic unity of the islands and atolls of the central Pacific was first documented in detail by Johann Reinhold Forster, a naturalist on James Cook’s second voyage of discovery to the Pacific (1772–1775). He suggested that the similarity of the languages spoken there, now known as Polynesian, reflected a comparatively shallow time-depth since their dispersal^1^. Forster’s seminal comparative study of Austronesian languages identified the lowland region of the Philippines in Island Southeast Asia (ISEA) as the ultimate source for the Polynesian languages and proposed a long-distance migration from there by the ancestors of today’s Polynesian speakers. This appeared to be the only explanation for the striking difference in phenotype that he observed between the peoples of the central Pacific and those of the intervening region, which is now known as Melanesia. Herein, the terms Melanesia and Micronesia are used in their geographical sense. We use the term Polynesia to include all islands and atolls whose inhabitants speak Polynesian languages, including 23 found throughout Melanesia and Micronesia, referred to as outlier Polynesia (Fig. 1a).

Separating the demographic histories of Polynesia and Melanesia became difficult to sustain with developments in archaeology during the second half of the 20th century. These established that the settlement of southern Melanesia (Santa Cruz, Vanuatu, New Caledonia and Fiji) and western Polynesia (Tonga, Samoa, Niue and Futuna) is marked by the same archaeological horizon, known as the Lapita Cultural Complex (LCC). The LCC first appears in northern Melanesia (the Bismarck Archipelago, Bougainville, and the Solomon Islands main chain) ~3,450–3,250 BP, and quickly spread into southern Melanesia ~3,200–3,000 BP, reaching Tonga and Samoa ~2,900 BP^2–4^. At the same time, the study of comparative linguistics has shown that the Oceanic branch of the Austronesian phylum of languages, of which Polynesian is a member, is spoken throughout most of Melanesia and parts of coastal New Guinea, and appears to be a recent intrusion from ISEA^5^. So while there is considerable overlap between the distributions of the LCC and the Oceanic languages, there remains a phenotypic divide between southern Melanesia and western Polynesia, which is observed between Fiji and Tonga^6,7^.

A central theme in this debate is the extent to which the development of the LCC involved local people in the Bismarck Archipelago of northern Melanesia^8–10^. An alternative is that the LCC represents the arrival of a largely pre-formed cultural package carried by speakers of proto-Oceanic languages from Taiwan, via the Philippines, in ISEA^11^. Hypotheses are placed on a continuum from a dendritic, radiating, phylogenetic model of cultural evolution that relies on the relative isolation of populations^12^, to one based on complex ongoing biological and cultural interaction between groups, leading to reticulated networks of genes and culture^9^. A compromise position has been promoted by the recognition of a Lapita homeland in the Bismarck Archipelago^10^, together with evidence that the genomes of contemporary Polynesians contain 20–30% ancestry typical of northern Melanesia and New Guinea^13,14^. This posits a period of limited cultural and genetic admixture involving migrants from ISEA during the early LCC phase in northern Melanesia ~3,450–3,250 BP^15^. Polynesian society then developed in relative isolation following the pioneering settlement of Tonga and Samoa ~2,900 BP^12^.

Genetic evidence for this intermediate model is provided by the presence of members of Y chromosome haplogroup (hg) C2a-M208, together with its daughter lineage C2a1-P33, among Polynesian speakers^16,17^. This is seen as a proxy for male-mediated admixture from northern Melanesian and New Guinean sources into the gene pool of migrants from ISEA during the formative period of the LCC in the Bismarck Archipelago, prior to the settlement of southern Melanesia and western Polynesia^13,18^. In contrast, the near fixation in Polynesian speaking groups of the mitochondrial lineage B4a1a1 is seen as evidence of a predominantly ISEA maternal heritage^13,19^. Subsequent research, however, has shown that B4a1a1 is widespread throughout northern Melanesia^20^, including regions that show no evidence of autosomal admixture with people from ISEA^21^. Alternatively, therefore, hg B4a1a1 might also have been present in northern Melanesia before the emergence of the LCC^22,23^. Similar ambiguity now exists over the origins of paternal lineage C2a-M208, due to its presence in ISEA^24^ and rather low overall frequencies in the Bismarck Archipelago and coastal New Guinea^17^.

An important advance in this debate is the recovery of ancient genomic DNA from LCC contexts on Vanuatu (~2,900 BP) (*n* = 3) and post-Lapita Tonga (~2,500 BP) (*n* = 1), since the results indicate people with close to 100% ancestry related to an ISEA heritage^25^. These data show that some settlers of the LCC period appear to have transited northern Melanesia and New Guinea from ISEA without receiving any significant amounts of genetic admixture. A second major finding is that the 20–30% ancestry originating from northern Melanesia and New Guinea, detected in contemporary genomes from the eastern fringe of southern Melanesia and western Polynesia, appears to have arrived during the 2^nd^ millennium BP (1,900–1,200 BP). This result is consistent with post-LCC movements of people into southern Melanesia and western Polynesia, in a process of polygenesis, being responsible for the differences in phenotype observed between the two regions^6^.

The potential significance of this proposed post-LCC migration for the phylogenetic approach to cultural evolution cannot be overstated. This is because the model is based on an Ancestral Polynesian Society (APS) developing in a western Polynesian homeland during the mid 3^rd^ millennium BP, followed by a rapid settlement of eastern Polynesia ~2,200 BP^12^. The settlement of eastern Polynesia, however, has witnessed significant reductions in the earliest secure radiometric dates in recent years. These currently stand at ~950 BP and come from Rai’atea in the Leeward Society Isles^26,27^, thereby excluding the original calibration for the model and subsequent revisions to it^28^. The archaeology for the phylogenetic model can also be challenged because the evidence post 2,500 BP suggests isolation of Tonga and Samoa, rather than the interaction invoked for the development of Proto-Polynesian language^29^. By ~950 BP, society in western Polynesia was differentiated, both culturally and linguistically, indicating that, if this late chronology is accurate, the source population for eastern Polynesia was likely a regional group rather than the hypothetical APS^29,30^.

A central component of the original phylogenetic model is the long-standing sub-grouping of the Polynesian languages. The initial divergence of Nuclear Polynesian from the Tongic languages is followed by a second-order split, between Proto-East Polynesian (Rapa Nui, Marquesan and Tahitic) and the rest of the Nuclear Polynesian languages (Samoic and all the Polynesian outlier languages)^31^ (Fig. 1b, left-hand tree). This sub-grouping recognizes the separation of Tongic and Samoic but is difficult to reconcile with a settlement of eastern Polynesia commencing ~950 BP, since it necessitates the second-order split, involving Proto-East Polynesian, to occur up to ~1,200 years earlier. An alternative linguistic sub-grouping that places the East Polynesian languages together with those of the central northern outliers (east coast of the northern Solomon Islands) provides a potential solution for the apparent discordance between archaeology and language^32,33^ (Fig. 1b, right-hand tree). This also challenges the orthodoxy within Polynesian studies that eastern Polynesia was settled directly from Samoa^11,12,28^. For Kirch and Green^28^, Samoa is ancient Hawa’iki, the cradle of Polynesian culture. In contrast, for Wilson^32^ Hawa’iki represents the ancient name for the Leeward Society Isles, which are referred to as the cultural and spiritual hub of eastern Polynesia in oral histories of the region, from where other islands and atolls were settled^34^.

The Leeward Society Isles, therefore, are of central importance to understanding the reasons for these conflicting signals from archaeology and language. If the ancestors of the Leeward Society Islanders experienced the same episode of ancient admixture as people in western Polynesia and outlier Polynesia during the mid 2^nd^ millennium BP^25^, this would support the late settlement chronology. In this study, we report the first genomic data from Bora Bora, Rai’atea and Taha’a, three of the Leeward Society Isles. We use the analysis of genotype and haplotype data to ascertain whether the signals of admixture present in these eastern Polynesian populations are similar to those from western and outlier Polynesia and identify potential donors to the ancestors of the Leeward Society Islanders. Further insights into the demographic history of eastern Polynesia is provided by the first deep re-sequencing of Polynesian Y chromosomes, complemented by high-resolution genotyping of key paternal and maternal lineages from the Leeward Society Isles and New Zealand.

## Results

### Data

Here we present a new genomic dataset sampled from the Leeward Society Islands, eastern Polynesia. We report high-resolution autosomal genotyping data from 30 individuals, complemented by genotyping and/or re-sequencing of uniparental loci (mtDNA and Y) from 81 individuals, including seven Y chromosomes re-sequenced by a target-capture method. In addition, we present new uniparental data from 49 Maori individuals sampled in New Zealand (Supplementary Tables S1–S3). The dataset is analyzed together with publicly available data from Island Southeast Asia, Melanesia and Polynesia (Supplementary Tables S1, S4 and S5). For detailed information about samples used in the present study, please refer to the Materials and Methods section.

### Autosomal analysi**s**

**Figure 2:**
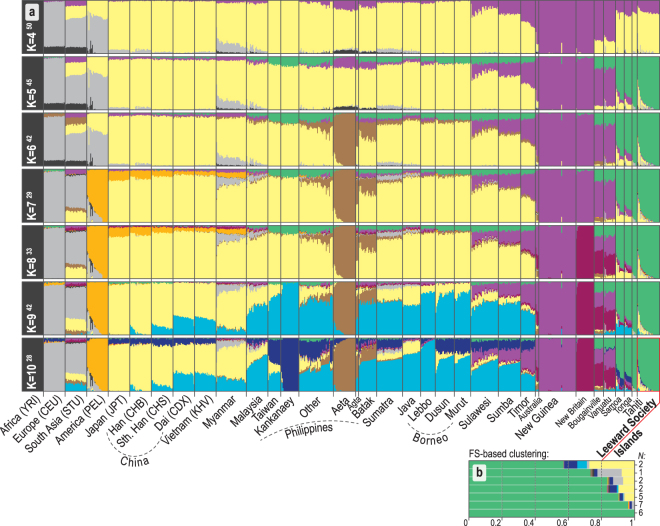
Ancestral genomic components in study populations estimated using ADMIXTURE. Details of the populations are provided in Supplementary Table S1B. The colors used have been selected to be equivalent to those used in Fig. 1. Only runs from *K* = 4 to 10 are shown, complete results (*K* = 2 to 15) are given in Supplementary Fig. S1. (**a**) For every value of K, the modal solution with the highest number (superscript) of ADMIXTURE^35^ runs is shown; individual ancestry proportions were averaged across all runs and the average cross-validation statistics were calculated across all runs from the same mode (Supplementary Fig. S2). The minimum cross-validation score is observed at K = 11 but no further components appear in the profiles of Polynesians after K = 10. Populations from the Philippines can be generally divided into Negritos (Aeta, Agta and Batak), Kankanaey of northwestern Luzon, and all others representing an amalgamation of groups from Luzon, Palawan and Visayas (see Fig. 1 and Supplementary Table S1B). (**b**) The average of K = 10 ADMIXTURE profiles for groups of Leeward Society individuals clustered by fineSTRUCTURE^37^ (Supplementary Fig. S6), indicating the heterogeneous distribution of East Asian and European ancestry among the Leeward Society Islanders.

The first two PCs of the principal components analysis (PCA, Fig. 1c) account for 38% of the variation in the studied dataset. The close overlap between eastern Polynesians and Samoans on the PC1 axis suggests similar amounts of genetic ancestry shared with New Guinea and northern Melanesia. The model-based analysis of autosomal SNPs using ADMIXTURE^35^ shows that, at *K* = 4, 70–80% of the Leeward Society Islander genomes can be characterized by the component typical of ISEA/East Asia (Fig. 2a); the remaining 20–30% of their genetic ancestry is best represented by Papuan speakers from New Guinea (light purple). From *K* = 5, Polynesians take their own ancestry (green), which, like their deflection on the PCA plot, is most likely due to genetic drift or, alternatively, cryptic relatedness or extreme inbreeding in studied populations. However, the latter is unlikely due to the lack of close relatives (up to third-degree, inclusive) in four Polynesian groups, and normal range of inbreeding coefficients when comparing to other human populations (*F*_*IS*_, Supplementary Table S6).

The lowest cross-validation (CV) score of ADMIXTURE is observed at *K* = 11, but no additional ancestries appear in Polynesians after *K* = 10, which has the second lowest CV score (Fig. 2a, Supplementary Figs S1 and S2). At *K* = 10, a dark blue component appears that is almost fixed in the Kankanaey of northwestern Luzon. The distinctive and uniform profiles of additional ISEA, Melanesian, and East Asian ancestries in two (Tonga and Samoa) out of four, otherwise very closely related, Polynesian groups hint that these may be the result of an old admixture process, rather than genetic drift, extreme bottlenecks or algorithmic artifacts. In contrast, the noticeably uneven distribution of the East Asian (yellow) and western European (grey) ancestry components within the profiles of the Leeward Society individuals (Fig. 2b) is consistent with recent historical admixture events (see haplotype-based admixture analysis below).

The outgroup *f*3^36^ allele-sharing plot shows the length of a phylogenetic branch shared between two study populations and African Yoruba. For the Leeward Society Isles (Supplementary Fig. S3, Supplementary Table S7), the *f3* allele-sharing results are consistent with a most recent evolutionary history shared with Samoa, Tahiti, and Tonga. It also suggests that the Kankanaey of the Philippines and Taiwanese aborigines are the next closest populations to all four Polynesian groups. These results remain robust to the different SNP subsets or population clustering schemes used in the present study (Supplementary Figs S3, S4, Supplementary Table S7). In contrast, the *f*3 admixture plots (Supplementary Fig. S5, Supplementary Table S7), which detect the presence of admixture in a study population from two reference groups, display different results for western and eastern Polynesia. These differences could be explained by a reduced effective population size for eastern Polynesians, caused by bottlenecks during the initial settlement process, or because Tonga and Samoa have experienced additional admixture since they last shared a common ancestral gene pool with Tahiti and the Leeward Society Isles.

**Figure 3:**
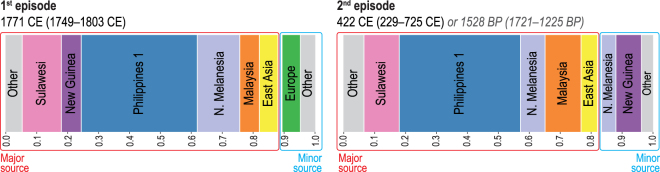
Population admixture events and inferred contact dates. Admixture events between genetic clusters obtained using fineSTRUCTURE^37^ (FS, Supplementary Fig. S6) and estimated with GLOBETROTTER^38^ (GT, Supplementary Table S8) for the Leeward Society Isles group. Each color represents a separate genetic cluster estimated with FS that acts as a donor to the recipient cluster (Leeward Society Isles) in the GT analysis. These donor groups are: Europe, East Asia, Malaysia, northern Melanesia (Bougainville), Philippines (‘Philippine 1’ cluster in FS), New Guinea and Sulawesi. ‘Other’ represents an amalgamation of groups contributing less than 3% ancestry to the admixture episodes in the genomes of Leeward Society Islanders. There is strong statistical support for two episodes of admixture; the ancient and recent events are represented by the left- and right-hand bar plots, respectively. Each episode involves two pairs of sources (minor and major); bar plots depict the inferred composition of the mixing sources for each, with admixture dates calculated using a generation time of 28 years. The dates for the older episode are given in the format of Common Era (CE) and Before Present (BP) for convenience. Detailed information about the inferred admixture episodes and composition of mixing sources is given in Supplementary Table S8.

The unsupervised fineSTRUCTURE (FS) analysis of haplotypes^37^ placed individuals into genetic clusters that include: Philippine groups from lowland Luzon, Palawan, and Visayas (‘Philippines 1’), Malaysia, Sulawesi, East Asia, northern Melanesia (Bougainville), New Guinea, and western Europe (Supplementary Fig. S6). The GLOBETROTTER (GT) analysis^38^ produced strong statistical support for two separate episodes of admixture involving the ancestors of the Leeward Society Islanders (Fig. 3, Supplementary Table S8). The first represents an average contribution of ~6% western European ancestry, which is dated to 1749–1803 CE. This is consistent with documented contact during Cook’s three voyages of exploration^1^, which took place 1768-71, 1772-75 and 1776-80. The second episode is estimated to have occurred in an interval from ~1,200 to 1,700 BP (229–725 CE), and is composed of a minor component (~17%), comprising mainly northern Melanesian and New Guinea sources, and a major one (~83%), in which the largest contributions are attributable to the ‘Philippines 1’, Sulawesi, and Malaysian clusters. The chronology indicates that this episode occurred prior to the earliest widely accepted radiometric dates for the permanent settlement of eastern Polynesia, which centre on ~950 BP and come from archaeological sites on Rai’atea in the Leeward Society Isles^26,27^. In addition, the presence of northern Melanesian ancestry in the minor component of the second (older) episode of admixture (~8% of the genome) reflects some genetic contact with this region for the ancestors of the Leeward Society Islanders prior to 1,200–1,700 BP.

In order to investigate the presence of the northern Melanesian contributions further, we performed a GT analysis on different subsets of Leeward Society Islanders as recipient groups (Supplementary Fig. S7, Supplementary Table S8). The results produced a spread of dates for the older episode of admixture, but always partitioned the northern Melanesian contribution into both sides of the admixture episode with point estimates ranging between *ca* 1,200 and 1,850 BP. Some of the variability in the dating may be due to the heterogeneous distribution of what appears to be recent admixture with people of East Asian ancestry (Figs 2B, S7, Supplementary Table S8), but the result is robust to variations in the makeup of the recipient group. This result, therefore, provides important evidence for either a period of migration from northern Melanesia into the ancestors of eastern Polynesians during the 3^rd^ millennium BP, or a process of biological admixture taking place during the LCC period in northern Melanesia prior to the pioneering settlement of Polynesia ~3,000 BP.

A further insight from the FS and GT analyses of haplotypes is the clear delineation between possible donor groups within the Philippine palette of populations. This excludes the Aeta, Batak, and Kankanaey clusters from any significant contribution to the population ancestral to the Leeward Society Isles (Figs 3, S7, Supplementary Table S8). The Philippine populations from the regions of Luzon, Palawan, and Visayas form a ‘Philippines 1’ cluster, which contributes nearly 40% of the genome of the Leeward Society Islanders. The apparent discrepancy with the analysis of unlinked SNPs (Supplementary Figs S3 and S4), which indicates the Kankanaey as being closest to the Leeward Society Isles, may be caused by the two methods measuring different aspects of the underlying genetic structure. In addition, ascertainment bias inherent to genotyping arrays data can affect the allele-sharing statistics. The GT analysis, in contrast, is based on combinations of linked markers, and is consequently more powerful and robust for identification of complex admixture events^38^.

### Uniparental haploid loci: mtDNA

Ninety-six percent of Leeward Society Isles mitochondrial lineages belong to the haplogroup (hg) B4a1a1 typical of Polynesian speaking populations. A PCA plot based on frequencies of mtDNA B4a1a lineages (Supplementary Fig. S8) places the Leeward Society Islands closest to Ontong Java (central northern Polynesian outlier, Fig. 1a) with the major western Polynesian populations of Tonga and Samoa among the most distant from eastern Polynesians. The Bayesian estimate of the time to the most recent common ancestor (MRCA) for well-supported clades of mitochondrial hg B4a1a1 (Supplementary Table S10A) is consistent with more than a third being significantly older than the first settlement of southern Melanesia and western Polynesia. The diversity-based age for B4a1a1 among Polynesian-speaking groups at ~5,700 BP (4,100–7,700 BP) is substantially older than the age of the LCC in northern Melanesia.

### Uniparental haploid loci: Y chromosome

The major Y chromosome haplogroup in the Leeward Society Isles is C2a1-P33 (67%), a sub-clade of C2a-M208, as it is throughout eastern Polynesia^16^, including the New Zealand Maori (52%), and the central northern outlier of Ontong Java (Supplementary Table S9B). Many of the haplotypes from eastern Polynesia (Leeward Society Isles, Tahiti, New Zealand Maori), and Ontong Java, are found near to the root of the hg C2a-M208 phylogenetic network (Supplementary Fig. S9). The PCA of this haplogroup and its sub-clades, including C2a1-P33, places the Leeward Society Islanders in closest overall proximity to individuals from the central northern Polynesian outlier of Ontong Java rather than those from Tonga and Samoa (Supplementary Fig. S10). The MRCA of the four target-sequenced Society Isles Y chromosomes provides an age of ~2,100 BP for the hg C2a1-P33 (Supplementary Fig. S11, Supplementary Table S10B).

Another Y chromosome hg, O3a’i-P164, represents a possible ISEA contribution to male lineages among Polynesians and occurs in western Polynesia at significant levels (35%). In the Leeward Society Islands O3a’i-P164 has a frequency of 11% (Supplementary Table S9B). Seven of the eight individuals belong to the O3i-B451 clade, which so far has only been identified among Austronesian speakers in ISEA^39,40^. All of these seven also typed positive for the downstream B450 marker and share an MRCA at ~5,700 BP with a Sama-Bajaw individual from Sulawesi (Supplementary Fig. S11, Supplementary Table S3). They also carry a rare triplication event at DYS385, which is present among individuals, not genotyped beyond the ancestral positions of O3′7-M122 and O3′6-M324, from New Zealand (Supplementary Table S3), western Polynesia, Tikopia (southern Polynesian outlier) and Fiji (southern Melanesia)^17,41^. These individuals, therefore, likely belong to hg O3i.

The Y chromosome diversity of the Leeward Society Isles is completed by hg O1-M119 and hg O6a-JST002611, which are prevalent in Taiwanese aborigines and East Asia^42^, respectively, and hg S2a-P79 (formerly K3-P79) (see Supplementary Fig. S11 and its legend online, Supplementary Table S9B). The latter occurs on average at a frequency 6% in eastern Polynesia, western Polynesia, and Ontong Java (central northern Outlier). The available high-resolution STR data place the S2a-P79 haplotypes of the Leeward Society Isles in close proximity to those from New Zealand Maori and Ontong Java (Supplementary Fig. S12).

## Discussion

The genomes of contemporary Polynesian-speaking groups appear to be a mosaic of components derived from the coming together of long-diverged sources from ISEA and the region of northern Melanesia/New Guinea^13,14,25^. How this came about is the subject of considerable debate^9,11,12,30^. Our haplotype-based analysis of high-density autosomal SNPs indicates that, for the ancestors of the Leeward Society Isles, most of this admixture occurred during a period spanning ~1,200–1,700 BP. These genetic dates are nearly identical to those of a previous analysis that used a different method and amalgamated haplotype data from western (Tonga) and outlier (Rennell, Bellona and Tikopia) Polynesia^25^. They contrast with older dates obtained using different data sets and methods, which vary from ~7,000 BP to ~2,700 BP^13,14,43^. The method used here has been demonstrated to accurately identify known historical admixture events during the past 2,000 years^38,44^, but it is also possible that other analytical approaches may provide insights into a different part of the genealogical process.

The presence of this demographic signal in the data from the Leeward Society Isles is important, since it is consistent with archaeological evidence for a late settlement model for eastern Polynesia ~950 BP, and, therefore, the linguistic sub-grouping of Wilson^32^ (Fig. 1b). The substantial body of linguistic evidence supporting this sub-grouping includes over 200 lexical and grammatical innovations that are shared between the languages of eastern Polynesia and the central northern outliers (Luanguia, spoken on Ontong Java, Takuu, Nukumanu and Nuguria). Moreover, these innovations are stepwise and directional in nature, a pattern that is only consistent with a west-to-east movement of people, tracing the origins of eastern Polynesians to central northern outlier Polynesia, rather than Samoa^32,33^. The principal component analysis and phylogenetic reconstruction of the Polynesian mtDNA B4a1a1 sub-groups and C2a1-P33 paternal lineages (Supplementary Figs S8–S10, S12), are consistent with this linguistic evidence for the recent settlement of eastern Polynesia from the central northern outliers.

A further important contribution to the debate on Polynesian origins is the partitioning of northern Melanesian ancestry into both sides of the admixture episode taking place ~1,200–1,700 BP in the ancestors of the Leeward Society Islanders (Fig. 3). In particular, the contribution of ~8% of this ancestry to the side containing the ISEA sources is significant, because it suggests an earlier episode of admixture affecting the population ancestral to the Leeward Society Islanders. This result is robust to analysis by subsets of the data (Supplementary Fig. S7), but it is not possible to determine how and when this northern Melanesian ancestry entered into the ancestral gene-pool of the Leeward Society Islanders. It, therefore, remains feasible that, for some groups of Austronesian speaking migrants from ISEA, genetic admixture accompanied cultural interaction during the formative period of the LCC in the Bismarck archipelago ~3,450–3,250 BP^8,15^, which precedes the settlement of southern Melanesia and western Polynesia by at least 200 years^3,45^.

The position of the Kankanaey as the closest group to the Leeward Society Islanders in the outgroup *f3* allele-sharing plots (Supplementary Figs 3 and 4), while not making any significant contribution to their genomes in the GLOBETROTTER^38^ (GT) results (Fig. 3) is potentially very revealing. It is arguable that one or other result is misleading as an effect of severe genetic drift. However, this hypothesis requires the concurrent excess retention of either SNPs (should *f*3 results be taken at face value), or haplotypes (should we trust only GT), typical of those found in the Leeward Society Islands today, which is statistically unlikely. Alternatively, while the Kankanaey are indeed the single best remaining proxy for the ancestors of the Leeward Society Islanders, the ‘Philippine 1’ cluster is admixed with a genetically closer population for those ancestors (comparing to the Kankanaey). Specifically, although the ‘Philippine 1’ cluster has received extensive admixture with other groups, which lowers their *f3* score, they retain the best proxy for the haplotypic variation found in the original ancestors of the Leeward Society Islanders. This hypothesis is preferred because the GT approach models the recipient population using donors who are reconstructed rather than observed, allowing for subsequent admixture in the donor groups^38^.

Within the geographical context of the Philippines, the GT results make sense because the populations making up the other three Philippines clusters are all located in mountainous regions and have languages that are either relics or indicate long-term isolation^46,47^. In contrast, the ancestors of the demographic expansion that led to the settlement of Polynesia are anticipated to be part of a recent seafaring tradition. This necessarily would have been based in the coastal regions and could be related to pre-existing trading networks within ISEA that already had links to Melanesia (see Donohue and Denham^48^ and comments for a discussion of this subject). In this respect, it is interesting to note that the age of the most recent common ancestor of the Y chromosome haplogroup O3i-B451 (5,900–8,100 BP, Supplementary Table S10B), proposed as a marker for the expansion of Austronesian speaking people throughout ISEA^40^, exceeds the proposed timing for the transfer of the Neolithic from Taiwan (4,200 BP)^11^.

Within the Society Islands themselves, maternally-inherited mitochondrial DNA lineages are strongly biased towards variants thought to be associated with the dispersal of Austronesian speakers (96% B4a1a1, Supplementary Table S9A). The best candidate for a contribution from the Austronesian speaking diaspora of ISEA to the male lineages of the Leeward Society Islands is haplogroup O3i-B451. However, it contributes less than 10% to the Leeward Society Islands paternal lineages (Supplementary Table S3). The majority of Y chromosome lineages have proposed ancestral associations with modern Papuan groups (C2a1-P33 and S2a-P79, Supplementary Table S9B)^13,17^. This sex bias holds across Polynesia and is observed as far back as Island Southeast Asia^49^, and may have resulted from the practice of exogamy and matrilocal post-marital residence among early Austronesian speaking groups^50^. A sex bias is also reflected in the nuclear genomes of Austronesian speakers and appears to be a characteristic of the Pacific region as a whole^25,51^.

In conclusion, the picture of Polynesian origins emerging from the present study is one of a more complex demographic history than that originally envisioned in the phylogenetic model of cultural evolution^12^. The results presented here provide support for models based on inter-connectivity among, and within, the different parts of the Pacific, rather than their relative isolation^8,9^. The new data concur with a late chronology for the settlement of eastern Polynesia, which fits better with the linguistic arguments and haploid data linking this region to the northern central Polynesian outliers. With respect to the ultimate origin of the Island Southeast Asian ancestry found in the Leeward Society Isles, the results indicate a significant role for the lowland region of the Philippines, as predicted by Johann Reinhold Forster in his seminal comparative study of languages conducted more than two hundred and forty years ago.

## Materials and Methods

### New samples

Thirty-six samples from the Leeward Society Islands were previously reported for Y chromosome genotypes^52^ and 44 new samples are reported here, making a total of 81 male individuals from three islands: Bora Bora (*n* = 14), Rai’atea (*n* = 36), Taha’a (*n* = 31). In addition, 49 male Maori individuals sampled in New Zealand are reported for Y chromosome genotypes (Supplementary Table S1A). All samples were collected with informed consent and with the approval of the institutional review boards at the University of Colorado, U.S.A., and La Trobe University, Melbourne, Australia. All experiments were performed in accordance with the relevant guidelines and regulations of the collaborating institutions.

### mtDNA analysis

The DNA extracts of 81 Leeward Society Islanders (Supplementary Table S1A) were genotyped for membership of mitochondrial haplogroups typical of eastern Polynesia^53–55^. Nomenclature followed that of Phylotree.org, mtDNA tree Build 17^56^ (Supplementary Table S2). This resulted in 78 individuals allocated to the hg B4a1a1 and three individuals to hg Q. The control region (nps 57–372 and nps 16024–16526) was sequenced for 36 samples that could not be allocated to the known sub-clades of these two haplogroups, and 25 samples were further selected for complete mitochondrial genome sequencing. Using information from the full sequences, additional nucleotides were typed by Sanger sequencing to complete the haplogroup assignment.

The 25 newly generated complete mtDNA sequences were merged with published data (see Supplementary Table S4), and a Bayesian phylogenetic approach in BEAST 1.8.4^57^ used to analyse two data sets comprising genomes belonging to hgs B4a1a1 (n = 442) and M29/Q (*n* = 111), respectively (Supplementary Table S4). The data sets were partitioned into the D-loop, rRNA genes, and first, second, and third, codon positions of the 13 protein-coding genes. Each data subset was assigned an independent model of nucleotide substitution, selected using the Bayesian information criterion in PartitionFinder^58^. Four demographic models for the tree prior were compared: constant size, exponential growth, logistic growth, and Bayesian skyride coalescent^59^, together with two models of rate variation across lineages: strict clock and uncorrelated lognormal relaxed clock^60^. Marginal likelihoods were calculated using path sampling with 25 power posteriors, with samples drawn every 2 M MCMC steps across a total of 50 M steps. For the B4a1a1 analysis, the best combination was a strict clock with a logistic growth coalescent model. For the M29-Q analysis, the combination of strict clock and Skyride model is reported because the demographic model showed a clear change in population size.

To calibrate the estimate of the timescale, a normal prior for the mutation rate was specified (mean 2.14 × 10^−8^ mutations/site/year, standard deviation 2.87 × 10^−9^)^61^. The posterior distributions of parameters, including the genealogy, were estimated using MCMC sampling. Samples were drawn every 5,000 steps over a total of 50 M MCMC steps. To check for convergence, each analysis was run in duplicate. After checking for acceptable MCMC mixing, the samples from the two runs were combined. Sufficient sampling was checked by computing the effective sample sizes of all parameters, which were found to be greater than 200.

To make a principal component analysis of the haplogroup B4a1a data, 442 complete Polynesian and Melanesian mtDNA genomes used for the BEAST analysis (Supplementary Table S4) were combined with the additional complete or partially complete mtDNA sequences from Melanesia^54^ (*n* = 378), Hawaii^55^ (*n* = 159), New Zealand Maori^53^ (*n* = 20), and the remaining hg B4a1a1 haplotypes from Leeward Society Islands (*n* = 55). Haplotypes were assigned to sub-clades of the hg B4a1a by the HaploGrep2 software^62^ and manual inspection of sequences. The resulting haplogroup frequencies were used to produce a population level PCA in R^63^ (Supplementary Fig. S8).

### Y chromosome analysis

Eighty-one male individuals from the Leeward Society Islands were genotyped for Y chromosome haplogroup specific SNPs by Sanger sequencing in a hierarchical manner, including new branch-defining SNPs from sub-clades O3i-B451 and O3i-B450. Unless otherwise stated, all nomenclature follows that of Karmin, *et al*.^39^ to avoid potential confusion. The Y chromosomes of nine individuals belonged to haplogroups typical of Europeans (G, J, and R) and were not subject to further analysis. In addition, the Y chromosomes of 49 Maori males sampled in New Zealand were genotyped (Supplementary Table S1A). These samples were also hierarchically tested to a level of phylogenetic resolution equivalent to the main haplogroup level in the Leeward Society Islanders (Supplementary Table S3).

The 72 Leeward Society Isles samples with non-European Y chromosomes, together with the 49 Maori samples, were further genotyped for 23 Y chromosome short tandem repeats (Y-STRs; Supplementary Table S3). After merging with comparative data from other sources^16,17,39,41,42^ and excluding individuals with partial results, this produced a data set of 15 microsatellites and these were used it to construct phylogenetic networks of hgs C2a-M208 and K*-M9 using the reduced median algorithm with the software Network 4.6.1.1 software^64^ (Fluxus-Engineering). The same 15 microsatellites occurring on the C2a-M208 background were used to perform PCA in R^63^, in order to examine the relationship of eastern, western and outlier Polynesia populations for this key haplogroup within Polynesian Y chromosome diversity (Supplementary Fig. S10).

Next, seven individuals belonging to hgs C2a1-P33 (*n* = 4), K-M9 (*n* = 1), O3i-B450 (*n* = 1) and O6a-JST002611 (*n* = 1) were selected for target-capture re-sequencing using the BigY service (Gene By Gene Ltd) (Supplementary Table S1A). The paired-end reads were mapped to the GRCh37 human reference sequence. The reconstruction and rooting of the phylogeny of the seven samples from the Leeward Society Islands used 56 sequences published in Karmin, *et al*.^39^ and 17 hg O individuals from the 1000 Genome Project^65^ (Supplementary Table S5). After filtering the data, the overlap between the data set was extracted and the ‘re-mapping filter’ based on modeling the poorly mapped regions was applied, as described in Karmin, *et al*.^39^. This resulted in 6.2 Mbp of usable sequence of the non-recombining male-specific Y chromosome region, and sites with minimum 95% call rate were used in the analysis.

A Bayesian phylogenetic approach in BEAST^57^ was used to analyse a final data set comprising 7669 SNPs from these 80 individuals. To correct for ascertainment bias, we added constant sites corresponding to the nucleotide composition across the remainder of the chromosome. The four demographic models and other details of the settings used in the MCMC analyses matched those used for analyses of mtDNA. The best-fitting model was exponential growth, which had a log Bayes factor of 8.079 compared with the next-best model (Bayesian skyride). To calibrate the estimate of the timescale, a mutation rate of 8.71 × 10^−10^ mutations/site/year^66^ was specified.

### Autosomal analysis

A set of 713,014 SNPs was screened using the HumanOmniExpress-24 BeadChip array in 30 individuals from the Leeward Societies Isles (Supplementary Table S1A). Twenty-six samples yielded high genotyping success rates (<5% missing genotypes), and 686,565 autosomal SNPs, with less than 5% missing data, were kept for further analyses. Inference of cryptic relationships between samples was performed using KING v. 1.4^67^ and no first-, second- or third-degree relatives were detected. A single sample clustered together with Europeans in the fineSTRUCTURE^37^ run (see below), and was excluded from all population level analyses (Supplementary Table S1B).

The study dataset was produced by merging newly generated Societies data with samples from mainland and ISEA, Melanesia, and Polynesia^44,68–73^, and with 25 random samples from multiple large continental reference populations from the 1000 Genomes Project^65^ (Fig. 1a, Supplementary Table 1B). Two independent datasets were produced. Firstly, a dataset comprised of 299,998 SNPs (after excluding SNPs with more than 5% missing data) from 570 samples was used for haplotype-based (fineSTRUCTURE, FS, and GLOBETROTTER^38^, GT) analyses, *f3* and *F*_*IS*_ statistics. FS/GT requires a much higher density of SNP coverage, which was not possible to achieve while keeping samples from Hudjashov, *et al*.^44^ due to the overlap between the different genotyping arrays used. Secondly, a dataset comprised of 92,972 SNPs (after excluding SNPs with more than 5% missing data) from 739 samples including those from Hudjashov, *et al*.^44^ was used for genotype-based analyses only (ADMIXTURE^35^, *f3*, *F*_*IS*_ and PCA) (Supplementary Table S1B). For PCA and ADMIXTURE, only unlinked SNPs with R^2^ < 0.2 were kept; 57,825 SNPs passed this criterion.

Although there is a substantial overlap between the two datasets used here (including populations from East and Southeast Asia, Philippines, Indonesia and Melanesia) some important differences need to be mentioned. The dataset used for the FS and GT analyses does not include samples from Taiwan, Tonga, Samoa and Tahiti. These four populations are, therefore, only in the dataset used for allele-frequency based analyses. However, the Kankanaey of northwestern Luzon in the Philippines are proposed as a proxy for early Austronesian speakers from Taiwan^71^. Polynesian populations from Hudjashov, *et al*.^44^ (Tonga, Samoa and Tahiti) were further controlled for the presence of cryptic relatedness between samples as described above by using the full SNP dataset from the original publication. In addition to the previously reported lack of first-degree relatives^44^, no other second- or third-degree relatives were found.

Maximum likelihood estimates of the ancestry of individuals were obtained with ADMIXTURE v. 1.30^35^. Following Cox, *et al*.^74^, fifty randomly seeded runs were performed for each number of ancestral populations (*K* = 2 to *K* = 15), and the results for each *K* were summarized with CLUMPP v. 1.1.2^75^. Runs with symmetric similarity coefficient > 0.9 were assigned to the same modal solution, and individual ancestry proportions were averaged across runs belonging to the same mode. The most frequent modal solution is reported.

Autosomal PCA was performed with the smartpca function of EIGENSOFT v. 3.0^76^ with no outlier removal step. *F*_*IS*_ (a measure of inbreeding) was calculated in Genepop v. 4.7.0^77^.

A series of *f3* tests were performed with ADMIXTOOLS v. 4.1^36^. Firstly, standard *f3* statistics were used as a formal test for admixture between all possible combinations of populations in the comparative dataset. Secondly, the outgroup *f3* test was implemented as a measure of the shared branch length between each of Polynesian groups and all other populations. For outgroup *f3*, the Yoruba population (YRI) from Africa was employed as the outgroup.

To assess the potential bias introduced by two different SNP subsets and sample clustering procedures used here, *f3* and *F*_*IS*_ were calculated as follows: (a) using the dataset of *ca* 93k SNPs and 739 samples (with data from Hudjashov, *et al*.^44^) and the original population affiliation; (b) using a dataset of *ca* 300k SNPs and 570 samples (without data from Hudjashov, *et al*.^44^) and the original population affiliation; (c) as per the approach outlined in (b), but using FS-based population grouping (see below and Supplementary Table S1B).

In order to take advantage of the benefits gained from including linkage information when working with high-density genetic data, we employed the fineSTRUCTURE (FS)^37^, CHROMOPAINTER^37^ and GLOBETROTTER (GT)^38^ framework. Genotypes were first phased with SHAPEIT v. 2^78^ using the HapMap phase II b37 recombination map^79^. Sample were assigned to genetic groups using fineSTRUCTURE v. 2 with default parameters; 7.5 M MCMC iterations were performed in total. The population dendrogram produced by FS was manually inspected and samples were assigned to 21 individual groups.

After excluding a single Leeward Society Isles sample with a very high proportion of European ancestry, we inferred admixture with GT using the remaining combined Societies sample set (*n* = 25). To gain insight into the admixture variance within the Leeward Society Islands, we performed additional GT runs using the individual clades of the FS dendrogram. For the latter approach, only clades with a minimum of five samples were included, and in one case (‘Societies 3’) the clade was amalgamated with its closest direct neighbor to pass the sample-size threshold. In total, 20 out of 25 samples were used in the individual GT runs. GT analysis was performed following the ‘full’ algorithm protocol^38,44^, where each recipient Society genome could copy chunks from the genomes of all other non-Societies donor clusters. One hundred bootstraps were used to assess the statistical significance of the admixture event and uncertainty of the inferred dates. Admixture dates were converted to years using the formula (*x* + 1) * 28^38^, where *x* is the number of generations since the admixture event and the generation interval is 28 years^80^.

### Data Availability

The genotyping SNP and STR data for mitochondrial and Y chromosomal DNA generated during the current study are included in the published article and its Supplementary Information files. The complete mitochondrial genome sequences generated during the current study are available from GenBank (https://www.ncbi.nlm.nih.gov/genbank/) under the accession numbers MG244202–MG244226. The seven novel Y chromosome sequences are available from European Nucleotide Archive (https://www.ebi.ac.uk/ena) under the accession number PRJEB22729. The Autosomal data produced from 30 Leeward Society Islanders is available from the corresponding author on reasonable request.

## Electronic supplementary material

Supplementary Information

Supplementary Table Legends

Supplementary Table S1

Supplementary Table S2

Supplementary Table S3

Supplementary Table S4

Supplementary Table S5

Supplementary Table S6

Supplementary Table S7

Supplementary Table S8

Supplementary Table S9

Supplementary Table S10
