# Supplementary material for: Investigating the origins of eastern Polynesians using genome-wide data from the Leeward Society Isles
Source: Sci Rep. 2018 Jan 29;8:1823. doi: 10.1038/s41598-018-20026-8 (PMC5789021; doi:10.1038/s41598-018-20026-8)
Supplement: Supplementary file 1 — Supplementary Information [file 41598_2018_20026_MOESM1_ESM.pdf]

Investigating the origins of eastern Polynesians using genome-wide data from the Leeward Society Isles

Georgi Hudjashov[1,2,8], Phillip Endicott[3,8,*], Helen Post[2], Nano Nagle[4], Simon Y. W. Ho[5], Daniel J. Lawson[6], Maere Reidla[2], Monika Karmin[2], Siiri Rootsi[2], Ene Metspalu[2], Lauri Saag[2], Richard Villems[2], Murray P. Cox[1], R. John Mitchell[4], Ralph L. Garcia-Bertrand[7], Mait Metspalu[2], Rene J. Herrera[7]

[1] Statistics and Bioinformatics Group, Institute of Fundamental Sciences, Massey University, Palmerston North, Manawatu, 4442, New Zealand

[2] Estonian Biocentre, Tartu, Tartumaa, 51010, Estonia

[3] Department Hommes Natures Societies, Musée de l'Homme, 75016, Paris, Ile de France, France

[4] Department of Biochemistry and Genetics, La Trobe University, Melbourne, Victoria, VIC 3086, Australia

[5] School of Life and Environmental Sciences, University of Sydney, Sydney, New South Wales, NSW 2006, Australia

[6] Integrative Epidemiology Unit, School of Social and Community Medicine, University of Bristol, Bristol, BS8 2BN, United Kingdom

[7] Department of Molecular Biology, Colorado College, Colorado Springs, Colorado, 80903, USA

[8] These authors contributed equally to this work

* Correspondence and requests for materials should be addressed to P.E. (email: phillip.endicott@gmail.com)

Supplementary Figure S1. Ancestral genomic components in study populations estimated using ADMIXTURE[1]. Runs from *K*=2 to *K*=15 were performed. For every value of *K*, the modal solution with the highest number (shown in the parentheses above the plot) of ADMIXTURE runs is shown; individual ancestry proportions were averaged across all runs and the average cross-validation statistics were calculated across all runs from the same mode (Supplementary Fig. S2). The minimum cross-validation score is observed at *K*=11, followed closely by *K*=10. Populations from the Philippines can be generally divided into Negritos (Aeta, Agta, Batak), Kankanaey of northwestern Luzon, and all others representing an amalgamation of groups from Luzon, Palawan and Visayas (Supplementary Table S1B).

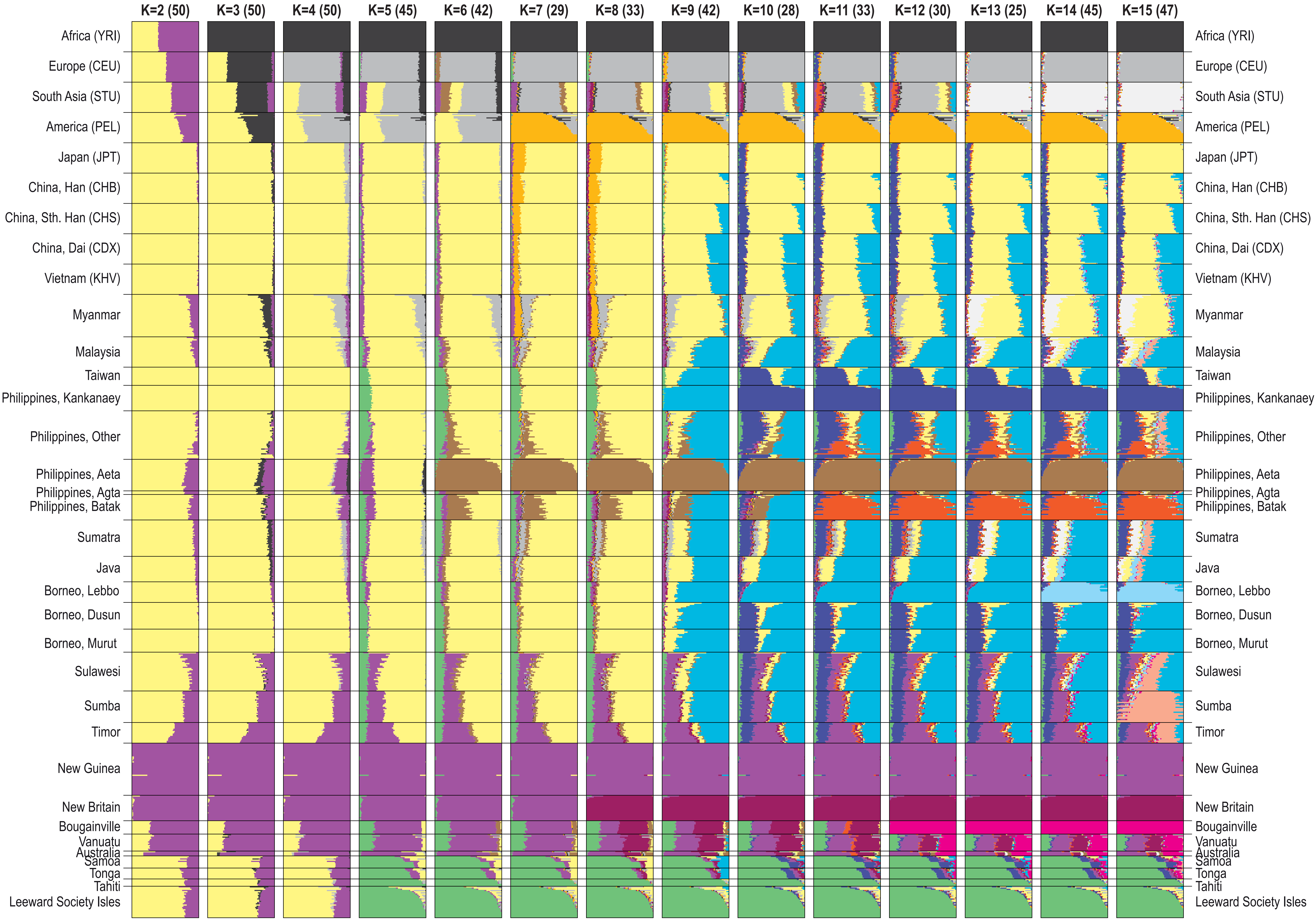
K=2 (50)
K=3 (50)
K=4 (50)
K=5 (45)
K=6 (42)
K=7 (29)
K=8 (33)
K=9 (42)
K=10 (28)
K=11 (33)
K=12 (30)
K=13 (25)
K=14 (45)
K=15 (47)
Africa (YRI)
Europe (CEU)
South Asia (STU)
America (PEL)
Japan (JPT)
China, Han (CHB)
China, Sth. Han (CHS)
China, Dai (CDX)
Vietnam (KHV)
Myanmar
Malaysia
Taiwan
Philippines, Kankanaey
Philippines, Other
Philippines, Aeta
Philippines, Agta
Philippines, Batak
Sumatra
Java
Borneo, Lebbo
Borneo, Dusun
Borneo, Murut
Sulawesi
Sumba
Timor
New Guinea
New Britain
Bougainville
Vanuatu
Australia
Samoa
Tonga
Tahiti
Leeward Society Isles

Supplementary Figure S2. Cross-validation (CV) scores from the ADMIXTURE analysis using the full data set.

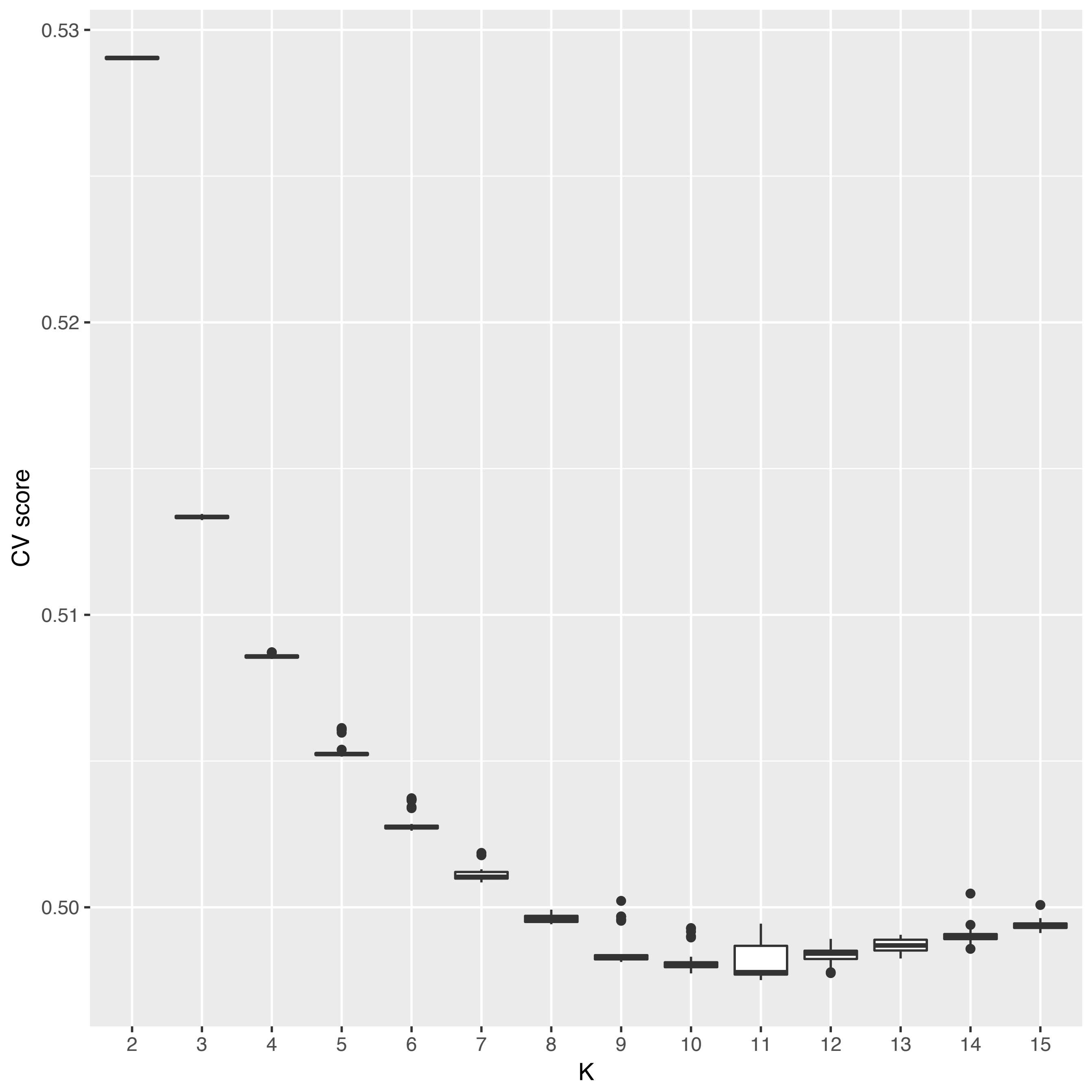

0.53
0.52
0.51
0.50
CV score
2
3
4
5
6
7
8
9
10
11
12
13
14
15
K

Supplementary Figure S3. Outgroup *f*3 allele-sharing plot between four Polynesian groups and reference populations. Dataset was comprised of 93k autosomal SNPs and 739 individuals, including data from Hudjashov, et al.[2]. The ((Polynesian target, reference population), YRI) test configuration was used. Detailed test results are given in the Supplementary Table S7D online.

Outgroup f3 analysis of four Polynesian populations, including samples from Hudjashov et al. (2017), 92,972 SNPs in total.

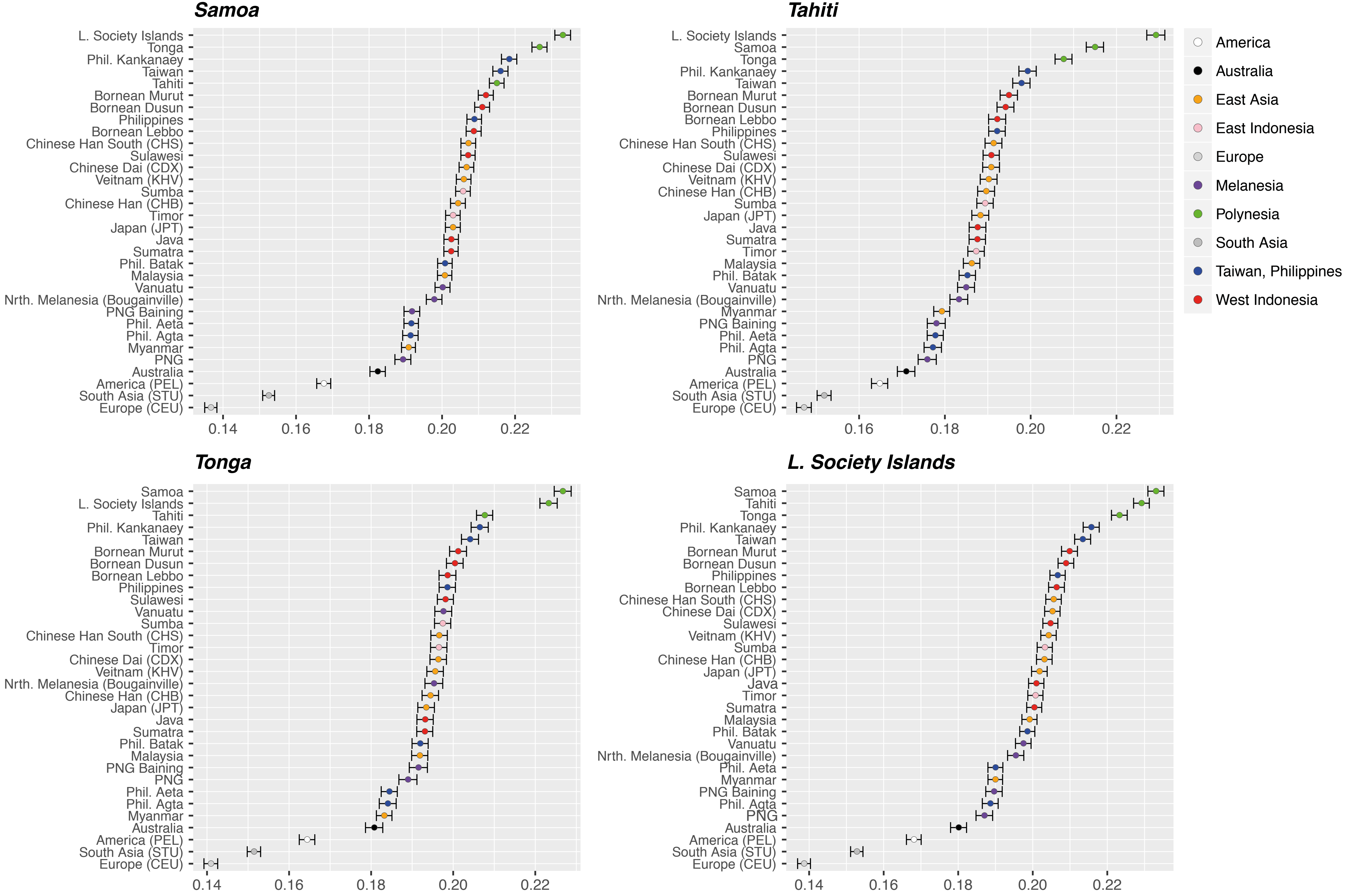

Supplementary Figure S4. Outgroup *f*3 allele-sharing plot between Leeward Society Islands and other reference populations. Dataset was comprised of 300k autosomal SNPs and 570 individuals, excluding data from Hudjashov, et al.[2]. The ((LSI target, reference population), YRI) test configuration was used. Detailed test results are given in the Supplementary Table S7E and S7F. Two different sample clustering schemes were used:

A) Samples were clustered according to fineSTRUCTURE results (Supplementary Fig. S6).
B) Samples were clustered using original population labels.

See Supplementary Table S1B online for cluster details.

Outgroup f3 analysis of samples from Leeward Society Islands, 299,998 SNPs in total. Excludes data from Hudjashov et al. (2017).

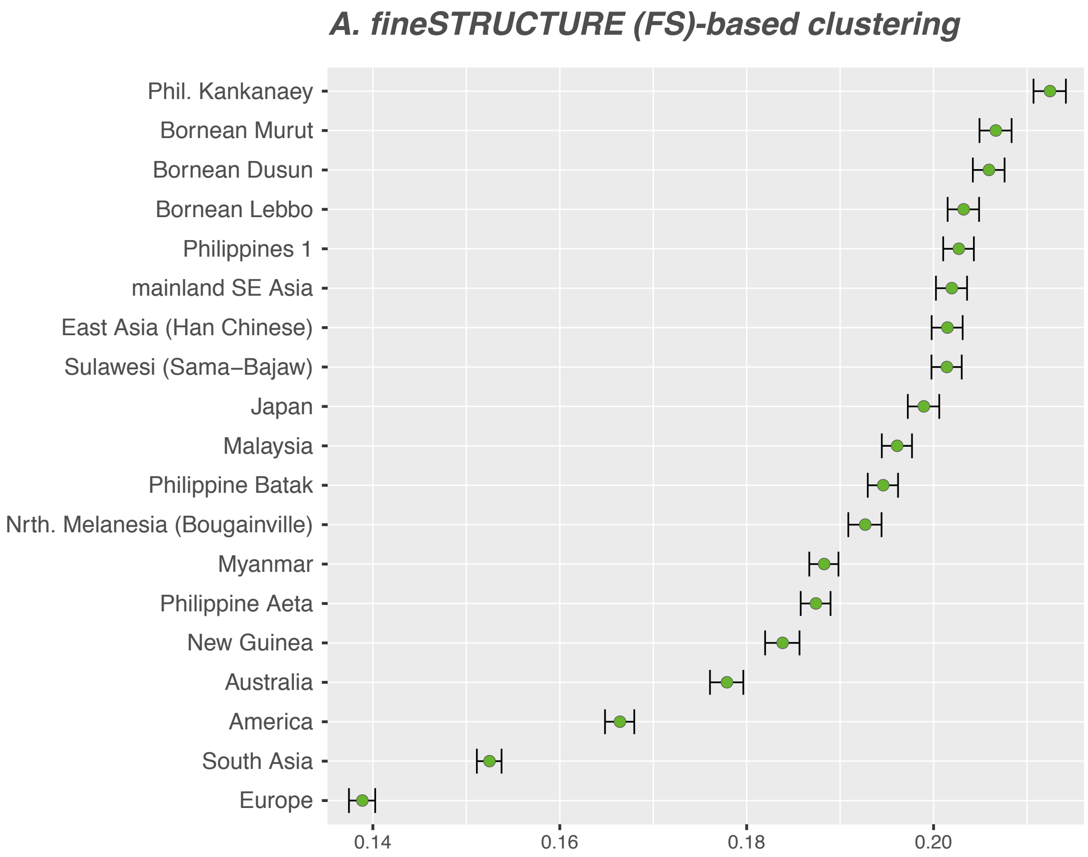

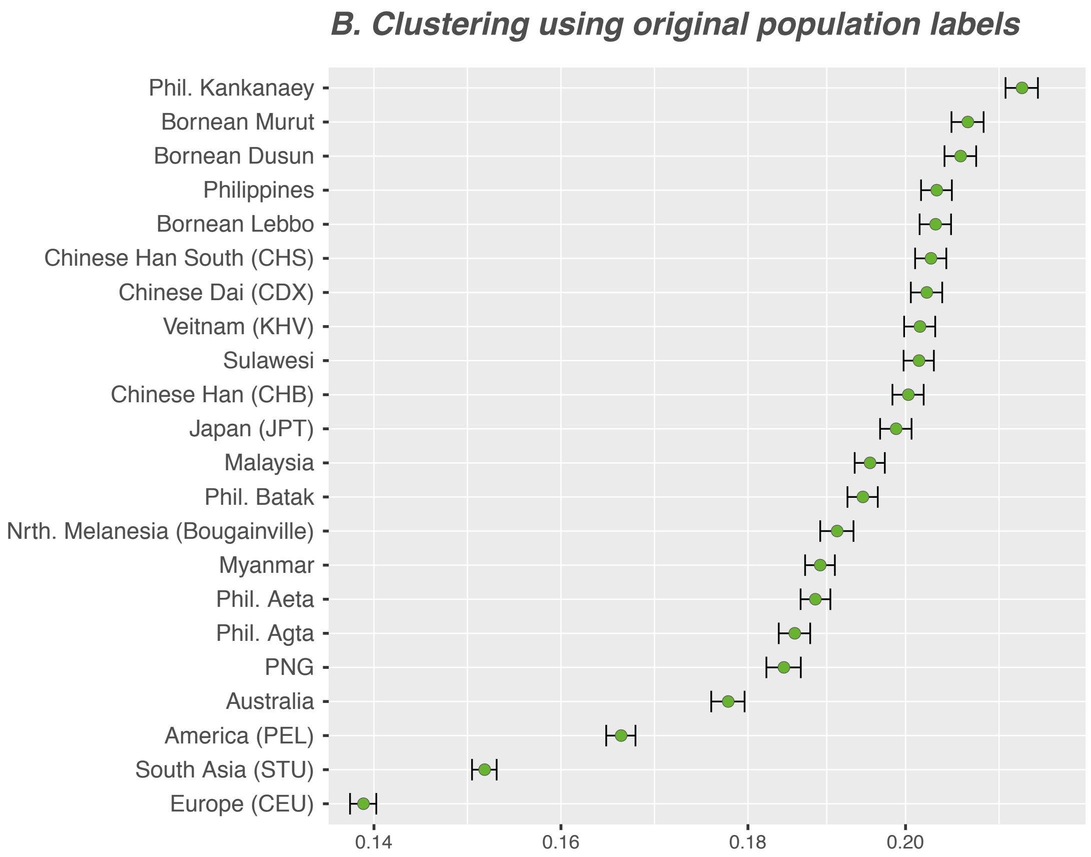

Supplementary Figure S5. *f*3 admixture analysis[3] for Tonga, Samoa, Tahiti and Leeward Society Islands. Dataset was comprised of 93k SNPs and 739 individuals, including data from Hudjashov, et al.[2]. All possible pairwise combinations of non-Polynesian reference populations were used to test for admixture in each Polynesian group individually. Reference pairs that returned significant results (negative *f3,* absolute z-score >2) in at least one Polynesian group are reported. Significant results are highlighted in orange. For full results refer to Supplementary Table S7A online.

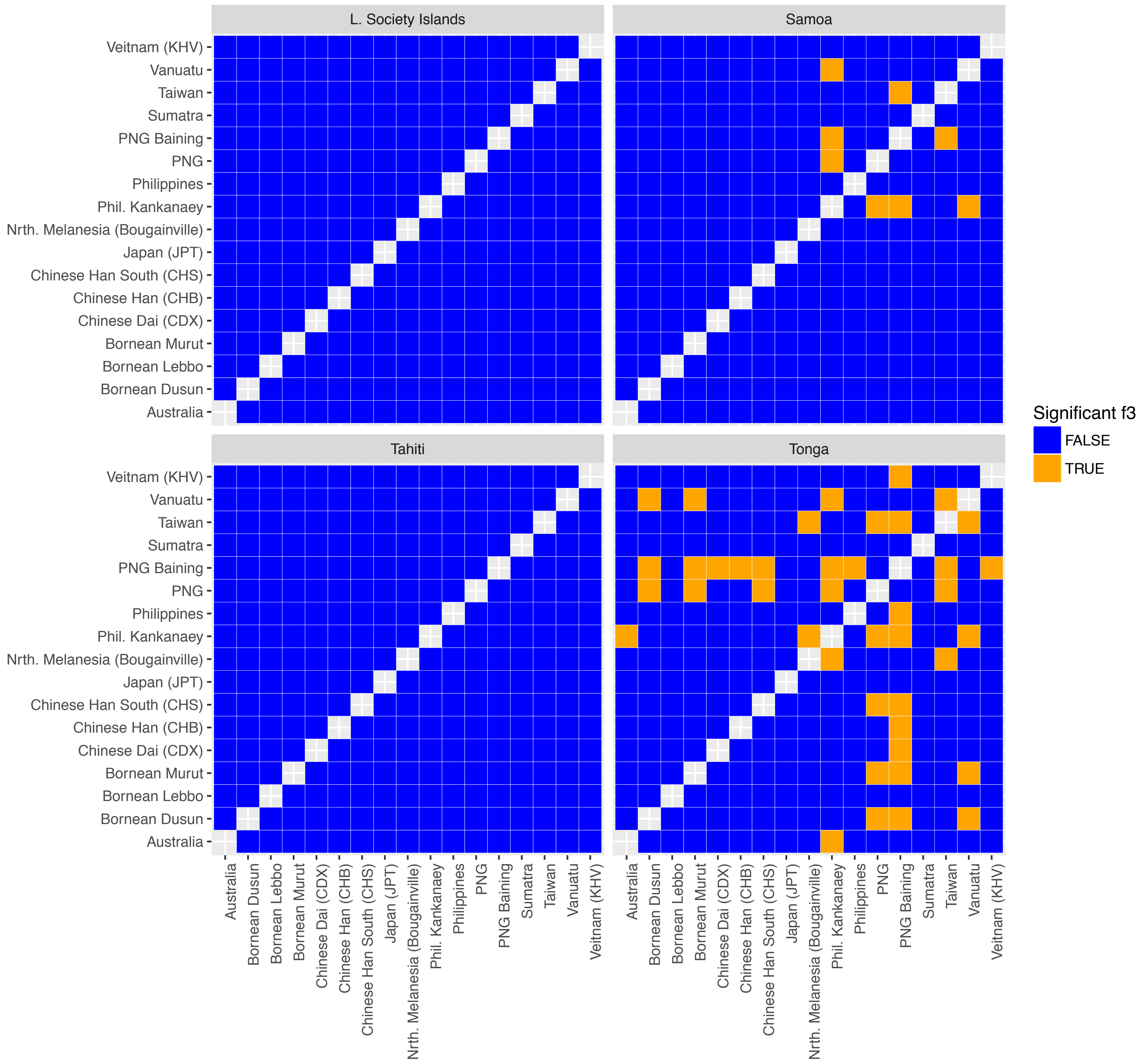
L. Society Islands
Samoa
Tahiti
Tonga
Veitnam (KHV)
Vanuatu
Taiwan
Sumatra
PNG Baining
PNG
Philippines
Phil. Kankanaey
Nrth. Melanesia (Bougainville)
Japan (JPT)
Chinese Han South (CHS)
Chinese Han (CHB)
Chinese Dai (CDX)
Bornean Murut
Bornean Lebbo
Bornean Dusun
Australia
Significant f3
FALSE
TRUE

Supplementary Figure S6. Clustering of individual samples from the comparative autosomal dataset, as inferred by fineSTRUCTURE (FS)[4]. The tree clusters individuals with similar copying vectors. Labels identify how many samples, and which ones, are included in each cluster. Individual tips were manually inspected further and grouped for the GLOBETROTTER (GT) analysis (Fig. 3, Supplementary Table S8); group names are shown on the left. Detailed sample affiliations to FS groups are given in Supplementary Table S1B online. A single Society islander with strong European admixture (strikethrough font) was excluded from the GT analysis. Three individual clades ('Society 3', 'Society 5' and 'Society 6') were used in additional GT runs (Supplementary Fig. S7, Supplementary Table S8) to gain insight into the admixture variance within the Leeward Society Islands.

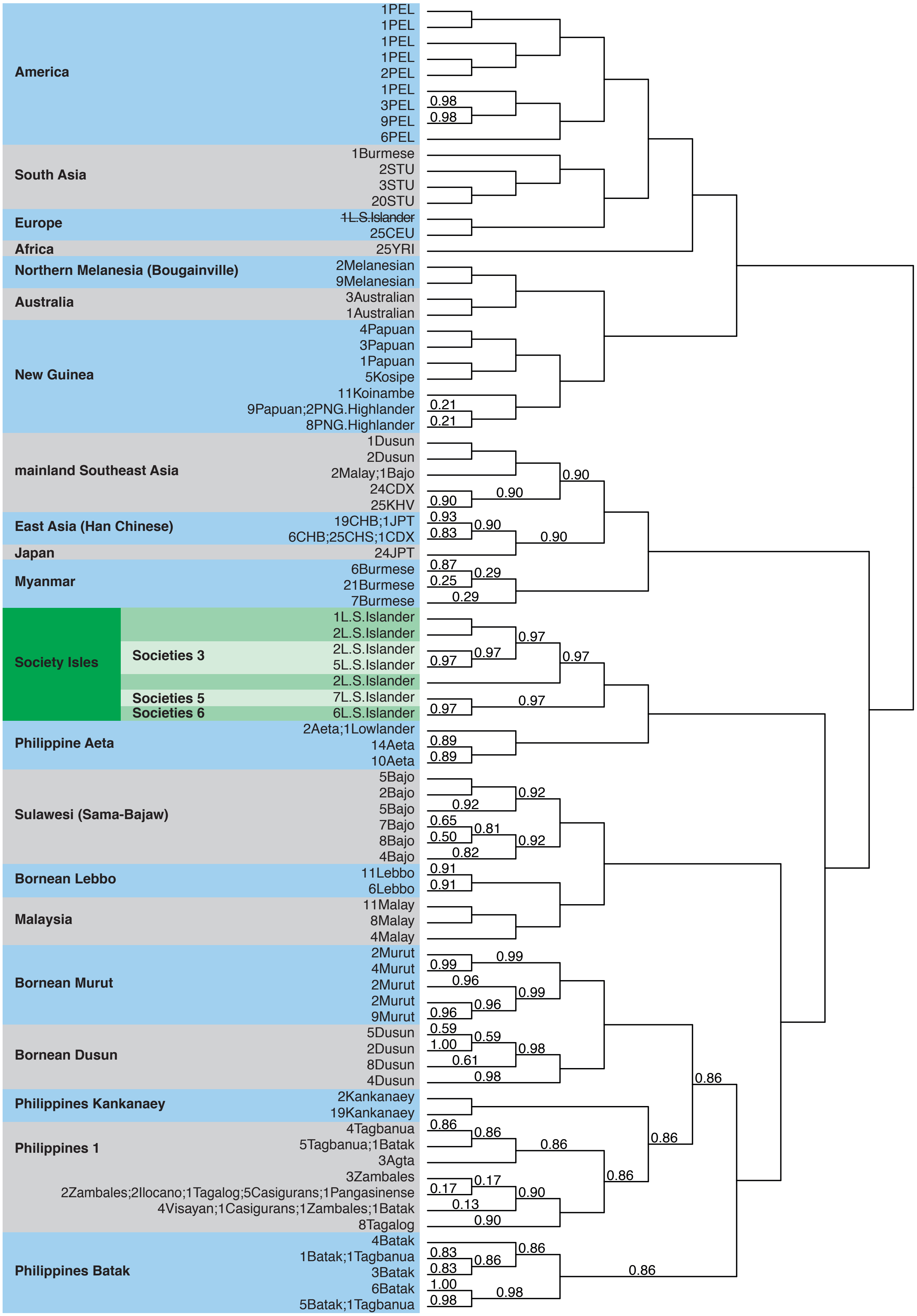
America
1PEL
1PEL
1PEL
1PEL
2PEL
1PEL
3PEL
9PEL
6PEL
South Asia
1Burmese
2STU
3STU
20STU
Europe
1L.S.Islander
25CEU
Africa
25YRI
Northern Melanesia (Bougainville)
2Melanesian
9Melanesian
Australia
3Australian
1Australian
New Guinea
4Papuan
3Papuan
1Papuan
5Kosipe
11Koinambe
9Papuan;2PNG.Highlander
8PNG.Highlander
mainland Southeast Asia
1Dusun
2Dusun
2Malay;1Bajo
24CDX
25KHV
East Asia (Han Chinese)
19CHB;1JPT
6CHB;25CHS;1CDX
Japan
24JPT
Myanmar
6Burmese
21Burmese
7Burmese
Society Isles
1L.S.Islander
2L.S.Islander
Societies 3
2L.S.Islander
5L.S.Islander
2L.S.Islander
Societies 5
7L.S.Islander
Societies 6
6L.S.Islander
Philippine Aeta
2Aeta;1Lowlander
14Aeta
10Aeta
Sulawesi (Sama-Bajaw)
5Bajo
2Bajo
5Bajo
7Bajo
8Bajo
4Bajo
Bornean Lebbo
11Lebbo
6Lebbo
Malaysia
11Malay
8Malay
4Malay
Bornean Murut
2Murut
4Murut
2Murut
2Murut
9Murut
Bornean Dusun
5Dusun
2Dusun
8Dusun
4Dusun
Philippines Kankanaey
2Kankanaey
19Kankanaey
Philippines 1
4Tagbanua
5Tagbanua;1Batak
3Agta
3Zambales
2Zambales;2Ilocano;1Tagalog;5Casigurans;1Pangasinense
4Visayan;1Casigurans;1Zambales;1Batak
8Tagalog
Philippines Batak
4Batak
1Batak;1Tagbanua
3Batak
6Batak
5Batak;1Tagbanua

Supplementary Figure S7. GLOBETROTTER[5] analysis of individual Leeward Society Islands clusters. Results for three individual clusters ('Society 3', 'Society 5' and 'Society 6') and combined sample are shown. For cluster details see Supplementary Fig. S6 and Supplementary Table S1B online. Each analysis detects two admixture events and for the older event there is always northern Melanesian ancestry in both the major and minor sources. The minor source of the older event is always made up of New Guinea and northern Melanesian ancestry.

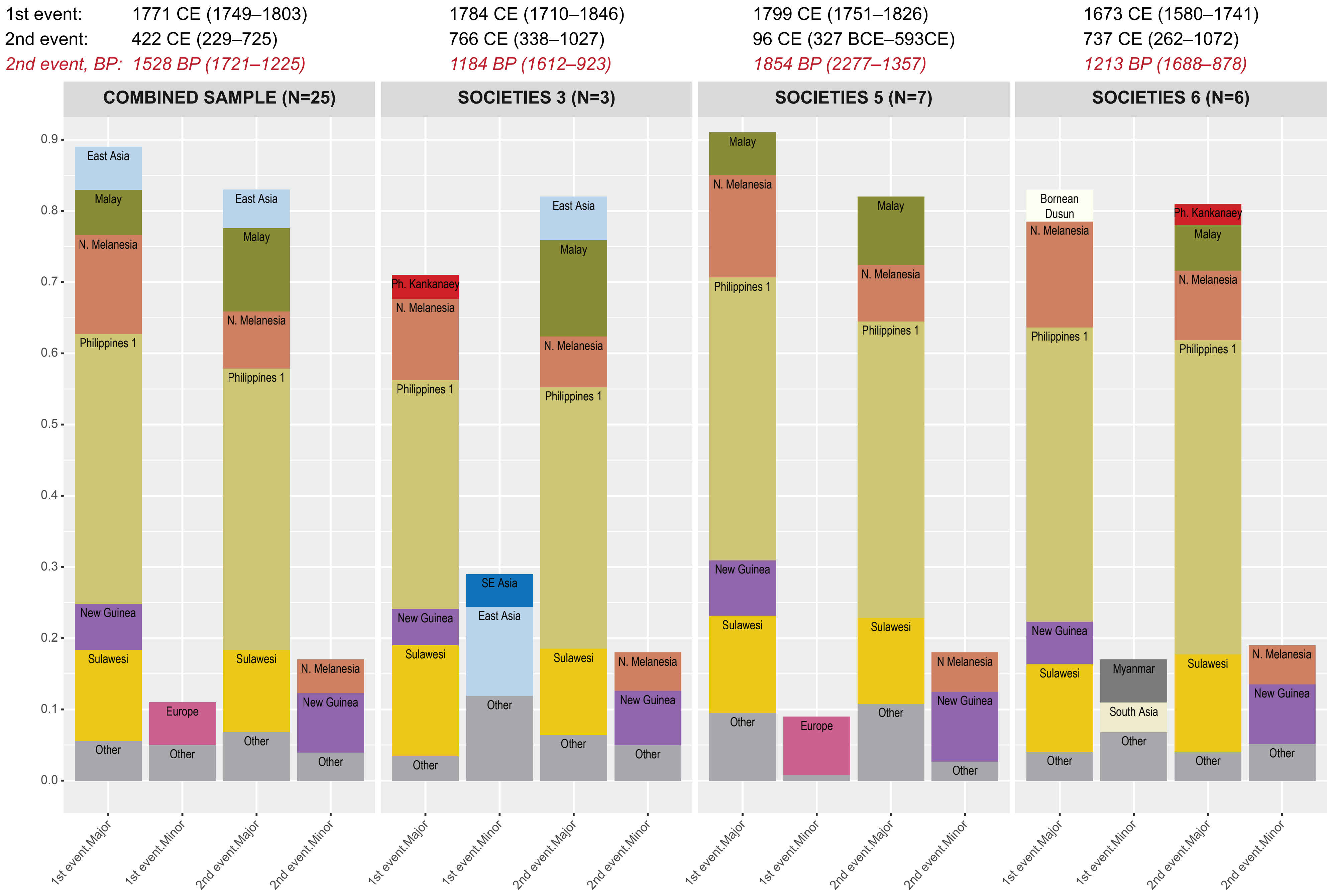
1st event: 1771 CE (1749–1803)
2nd event: 422 CE (229–725)
2nd event, BP: 1528 BP (1721–1225)
1784 CE (1710–1846)
766 CE (338–1027)
1184 BP (1612–923)
1799 CE (1751–1826)
96 CE (327 BCE–593CE)
1854 BP (2277–1357)
1673 CE (1580–1741)
737 CE (262–1072)
1213 BP (1688–878)
COMBINED SAMPLE (N=25)
SOCIETIES 3 (N=3)
SOCIETIES 5 (N=7)
SOCIETIES 6 (N=6)
0.0
0.1
0.2
0.3
0.4
0.5
0.6
0.7
0.8
0.9
East Asia
Malay
N. Melanesia
Philippines 1
New Guinea
Sulawesi
Other
Europe
SE Asia
Ph. Kankanaey
N Melanesia
Bornean Dusun
Myanmar
South Asia
1st event.Major
1st event.Minor
2nd event.Major
2nd event.Minor

Supplementary Figure S8. PCA based on the frequencies of sub-groups of the mitochondrial haplogroup B4a1a among Polynesian and Melanesian populations for which detailed genotyping information is available. These make up 96% of the maternal lineages in the Leeward Society Isles, 98% in outlier Polynesia and 94% in western Polynesia. Among the populations external to eastern Polynesia, the PCA plots place the Leeward Society Isles as the nearest neighbor to Ontong Java from the central northern outliers. The most distant Polynesian speaking groups are Samoa, Futuna, Tonga, and Tikopia the sole southern outlier group in the analysis. The most distant non-Polynesian speaking populations are consistently Gela, Guadalcanal and Savo in the southern Solomons. PCA was produced in R[6] using prcomp() function.

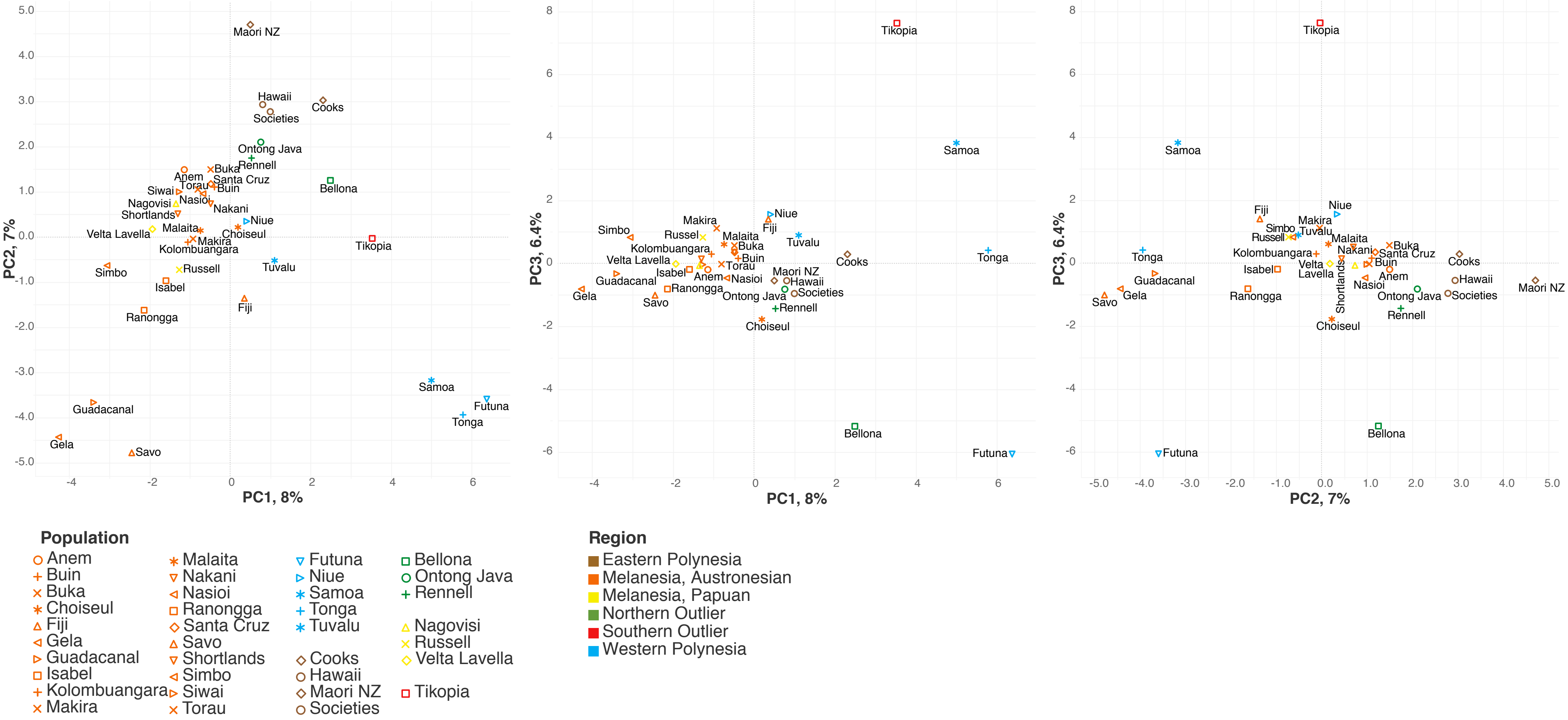

PC1, 8%
PC2, 7%
PC1, 8%
PC3, 6.4%
PC2, 7%
PC3, 6.4%
Population
Anem
Buin
Buka
Choiseul
Fiji
Gela
Guadacanal
Isabel
Kolombuangara
Makira
Malaita
Nakani
Nasioi
Ranongga
Santa Cruz
Savo
Shortlands
Simbo
Siwai
Torau
Futuna
Niue
Samoa
Tonga
Tuvalu
Cooks
Hawaii
Maori NZ
Societies
Bellona
Ontong Java
Rennell
Nagovisi
Russell
Velta Lavella
Tikopia
Region
Eastern Polynesia
Melanesia, Austronesian
Melanesia, Papuan
Northern Outlier
Southern Outlier
Western Polynesia

Supplementary Figure S9. **Reduced median STR network[7] using fifteen loci for hg C2a-M208 and C2a1-P33 individuals constructed with data from the current and previously published studies[8-11].** Node sizes are proportional to the number of individuals and the smallest node represents one person. The network includes two genomes from Sulawesi: C2a-M208 (GS000017004) and C2-M38 (GS000017005). The four Society Isles Y chromosomes sequenced by target-capture are indicated, which have a MRCA of ~2,100 BP. The approximate positions of the P33 and M208 markers are indicated. The C2a1-P33 individuals share a MRCA with GS000017004 (C2a-M208) at ~10,500 BP. The shaded area containing haplotypes from the Solomon Islands indicates that their position in the network is unstable, due to missing intermediate haplotypes.

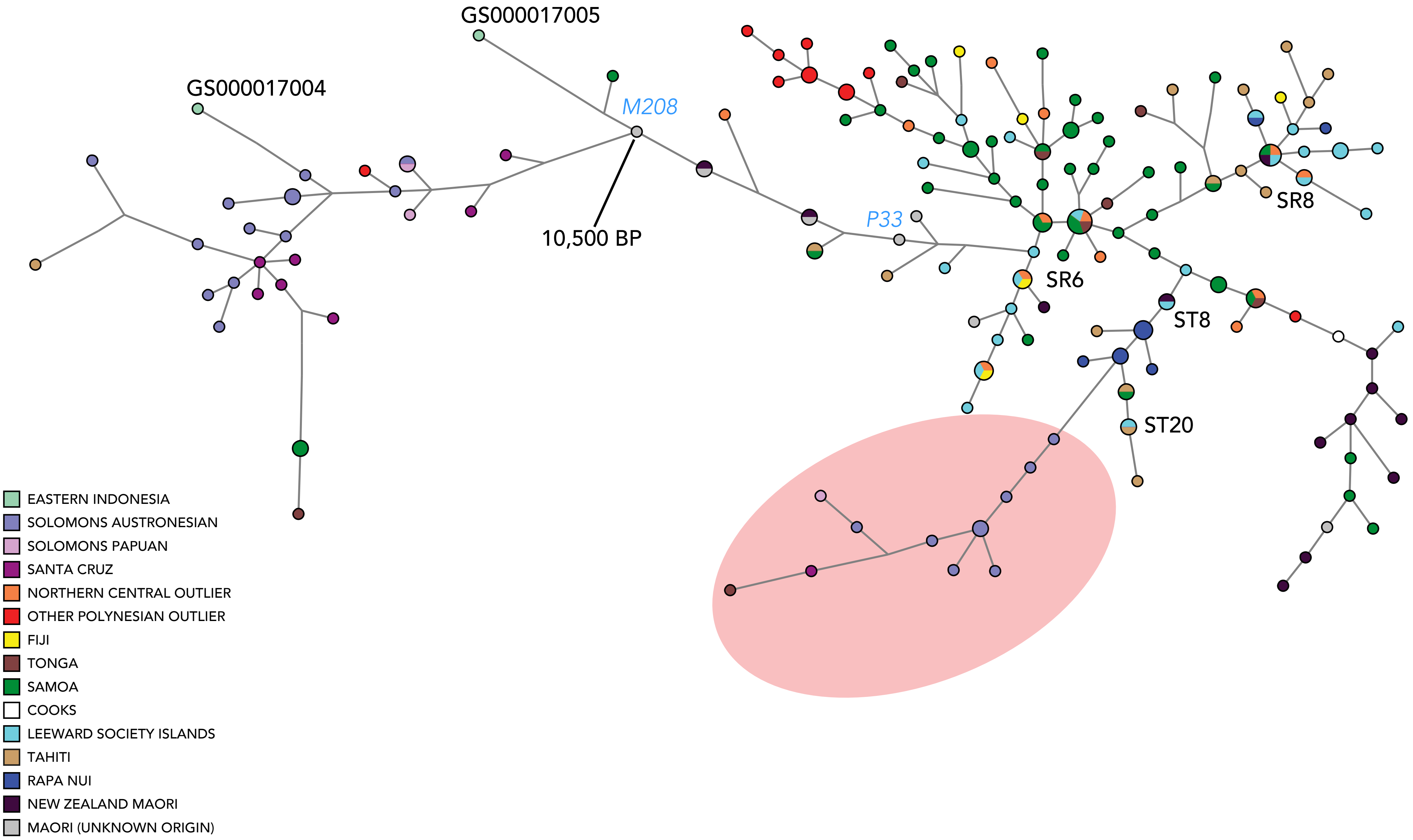
GS000017005
GS000017004
M208
10,500 BP
P33
SR6
SR8
ST8
ST20
EASTERN INDONESIA
SOLOMONS AUSTRONESIAN
SOLOMONS PAPUAN
SANTA CRUZ
NORTHERN CENTRAL OUTLIER
OTHER POLYNESIAN OUTLIER
FIJI
TONGA
SAMOA
COOKS
LEEWARD SOCIETY ISLANDS
TAHITI
RAPA NUI
NEW ZEALAND MAORI
MAORI (UNKNOWN ORIGIN)

Supplementary Figure S10. PCA representing the diversity at 15 STRs typed on individuals with Y chromosomes belonging to the haplogroup C2a-M208 among Polynesian speaking populations for which data is available. Of these groups, only the western Polynesian populations from Samoa and Tonga were not typed for the downstream marker C2a1-P33 but membership is inferred for all but one of these individuals (see Supplementary Fig. S9). The haplogroup C2a1-P33, which constitutes 68% of the lineages found in the Leeward Society Isles, and 56% of those in Ontong Java, has a very limited distribution outside of Polynesian speaking communities[8]. The PCA shows that the haplotypes found in the Leeward Society Isles share a substantial overlap with those from the northern outliers of Ontong Java and Rennell, while, with minor exceptions, plotting separately to those of western Polynesia. PCA was produced in R[6] using prcomp() function.

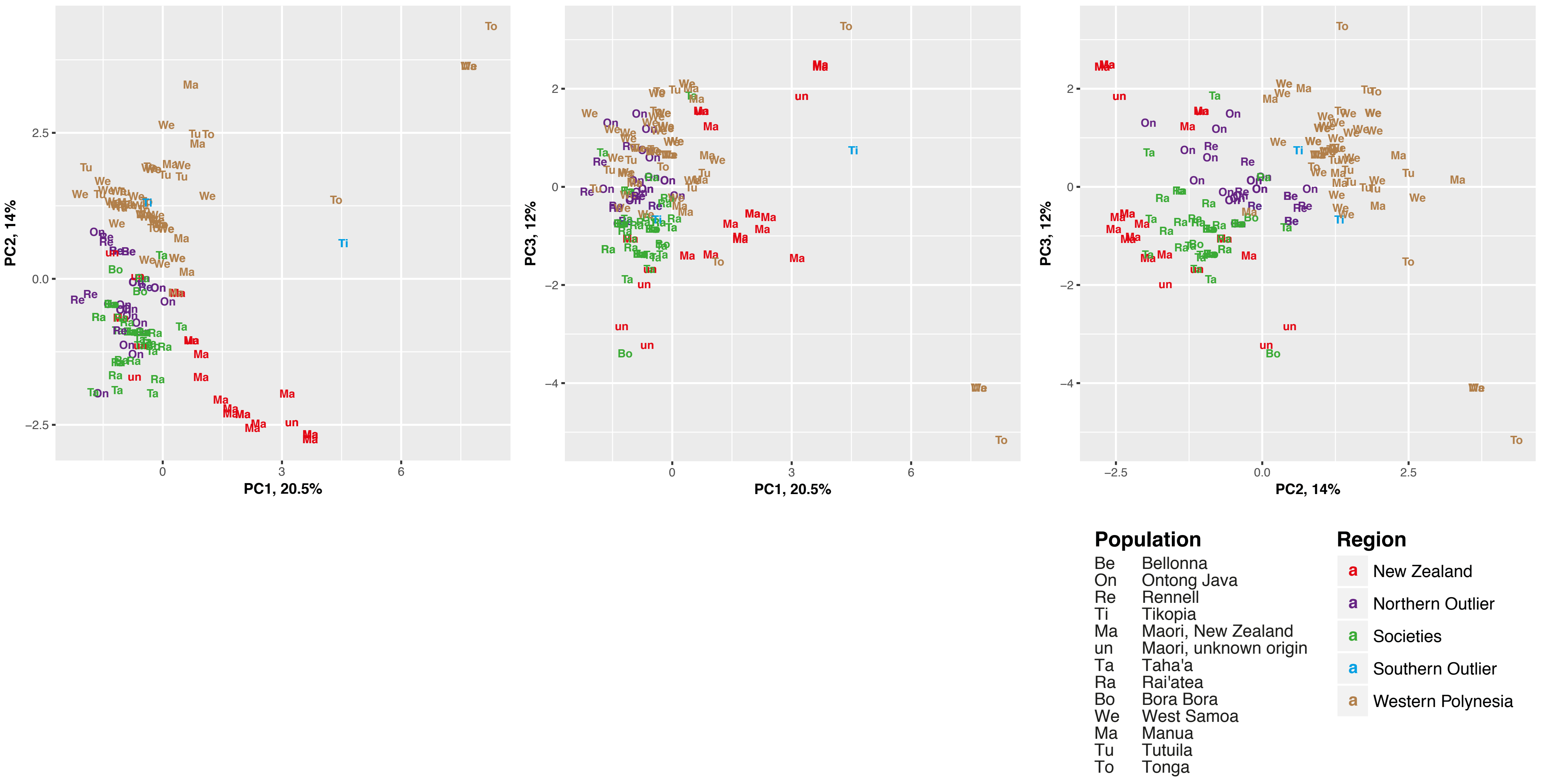
PC2, 14%
PC1, 20.5%
PC3, 12%
PC1, 20.5%
PC3, 12%
PC2, 14%
2.5
0.0
−2.5
0
3
6
2
0
−2
−4
−2.5
0.0
2.5
Population
Be Bellonna
On Ontong Java
Re Rennell
Ti Tikopia
Ma Maori, New Zealand
un Maori, unknown origin
Ta Taha'a
Ra Rai'atea
Bo Bora Bora
We West Samoa
Ma Manua
Tu Tutuila
To Tonga
Region
New Zealand
Northern Outlier
Societies
Southern Outlier
Western Polynesia

Supplementary Figure S11. Phylogenetic tree produced by the Bayesian analysis of Y chromosomes conducted with the BEAST software[12], using a data set comprising 7669 SNPs from 80 individuals (for details see Supplementary Table S5 online). All nodes on the tree have 100% support, except those that are indicated by numbers. The scale provides an indication of the ages associated with the 95% Higher Probability Distributions for each node, shown in grey shade.

For consistency with previous research, and to avoid potential confusion, the nomenclature of the tips is according to Karmin, et al.[9], with the following exceptions:

* All four Leeward Society Islander haplogroup C2a-M208 Y chromosomes also carry the P33 marker designating the C2a1 clade following the nomenclature of Delfin, et al.[8] and Cox, et al.[10] (shown in red italics). This nomenclature (C2a1-P33) was used in the present study. Transversions newly identified here, which are specific to the branch defined by P33 marker, are shown on the left-hand side of the tree.

** The exact phylogenetic position of P79 was previously unknown and chromosomes characterized by this SNP were only defined to the macro-haplogroup K level (K3-P79 in Delfin, et al.[8]). Our analysis of new high-coverage Y sequences from the Leeward Society Islands places this marker on a sister branch of the Bornean Lebbo Y chromosome from Karmin, et al.[9] (hg S2 in the original publication). According to the updated topology proposed here P79 now characterizes hg S2a (shown in green italics). Transversions specific to both the Leeward Society Islander from Rai'atea (SR22) and the Bornean Lebbo individual are shown on the left-hand side of the tree.

The estimated date of key nodes in the tree is given in Supplementary Table S10 online. Phylogeographic inference is limited by the availability of complete genomes for comparison but indication of the region and/or languages of interest are given by colour-coding of the tips. The blue colour indicates speakers of the Malayo-Polynesian branch of the Austronesian language family. The phylogeny of C2a-M208 does not currently indicate a region of origin; that of O3i-B451 strongly suggests an origin in Island Southeast Asia as indicated by present and other studies[13].

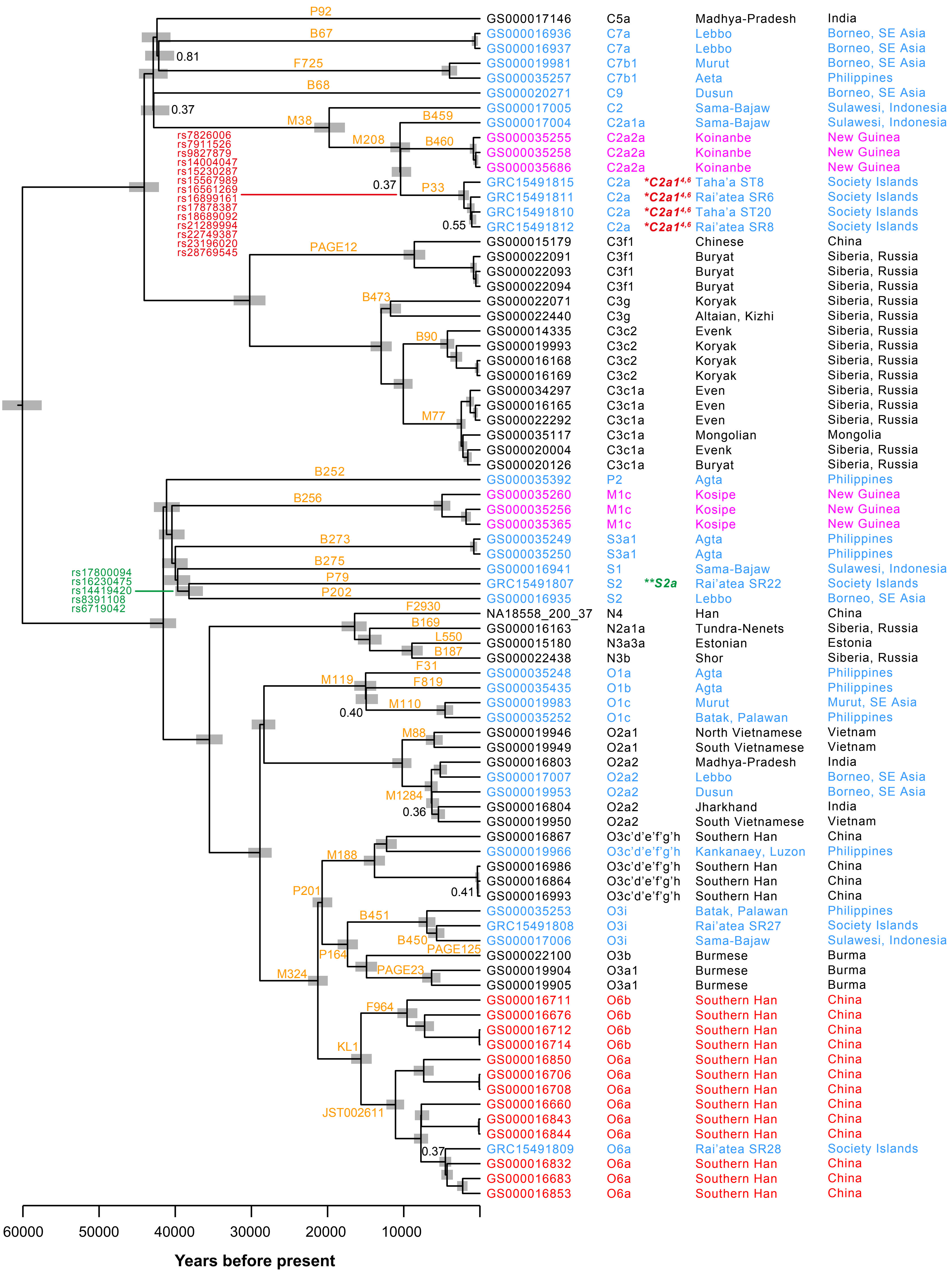
P92
B67
0.81
F725
B68
0.37
M38
B459
M208
B460
rs7826006
rs7911526
rs9827879
rs14004047
rs15230287
rs15567989
rs16561269
rs16899161
rs17878387
rs18689092
rs21289994
rs22749387
rs23196020
rs28769545
0.37
P33
0.55
PAGE12
B473
B90
M77
B252
B256
B273
B275
P79
P202
rs17800094
rs16230475
rs14419420
rs8391108
rs6719042
F2930
B169
L550
B187
F31
M119
F819
M110
0.40
M88
M1284
0.36
M188
P201
0.41
B451
B450
PAGE125
P164
PAGE23
M324
F964
KL1
JST002611
0.37
GS000017146 C5a Madhya-Pradesh India
GS000016936 C7a Lebbo Borneo, SE Asia
GS000016937 C7a Lebbo Borneo, SE Asia
GS000019981 C7b1 Murut Borneo, SE Asia
GS000035257 C7b1 Aeta Philippines
GS000020271 C9 Dusun Borneo, SE Asia
GS000017005 C2 Sama-Bajaw Sulawesi, Indonesia
GS000017004 C2a1a Sama-Bajaw Sulawesi, Indonesia
GS000035255 C2a2a Koinanbe New Guinea
GS000035258 C2a2a Koinanbe New Guinea
GS000035686 C2a2a Koinanbe New Guinea
GRC15491815 C2a *C2a1 4,6 Taha'a ST8 Society Islands
GRC15491811 C2a *C2a1 4,6 Rai'atea SR6 Society Islands
GRC15491810 C2a *C2a1 4,6 Taha'a ST20 Society Islands
GRC15491812 C2a *C2a1 4,6 Rai'atea SR8 Society Islands
GS000015179 C3f1 Chinese China
GS000022091 C3f1 Buryat Siberia, Russia
GS000022093 C3f1 Buryat Siberia, Russia
GS000022094 C3f1 Buryat Siberia, Russia
GS000022071 C3g Koryak Siberia, Russia
GS000022440 C3g Altaian, Kizhi Siberia, Russia
GS000014335 C3c2 Evenk Siberia, Russia
GS000019993 C3c2 Koryak Siberia, Russia
GS000016168 C3c2 Koryak Siberia, Russia
GS000016169 C3c2 Koryak Siberia, Russia
GS000034297 C3c1a Even Siberia, Russia
GS000016165 C3c1a Even Siberia, Russia
GS000022292 C3c1a Even Siberia, Russia
GS000035117 C3c1a Mongolian Mongolia
GS000020004 C3c1a Evenk Siberia, Russia
GS000020126 C3c1a Buryat Siberia, Russia
GS000035392 P2 Agta Philippines
GS000035260 M1c Kosipe New Guinea
GS000035256 M1c Kosipe New Guinea
GS000035365 M1c Kosipe New Guinea
GS000035249 S3a1 Agta Philippines
GS000035250 S3a1 Agta Philippines
GS000016941 S1 Sama-Bajaw Sulawesi, Indonesia
GRC15491807 S2 **S2a Rai'atea SR22 Society Islands
GS000016935 S2 Lebbo Borneo, SE Asia
NA18558_200_37 N4 Han China
GS000016163 N2a1a Tundra-Nenets Siberia, Russia
GS000015180 N3a3a Estonian Estonia
GS000022438 N3b Shor Siberia, Russia
GS000035248 O1a Agta Philippines
GS000035435 O1b Agta Philippines
GS000019983 O1c Murut Murut, SE Asia
GS000035252 O1c Batak, Palawan Philippines
GS000019946 O2a1 North Vietnamese Vietnam
GS000019949 O2a1 South Vietnamese Vietnam
GS000016803 O2a2 Madhya-Pradesh India
GS000017007 O2a2 Lebbo Borneo, SE Asia
GS000019953 O2a2 Dusun Borneo, SE Asia
GS000016804 O2a2 Jharkhand India
GS000019950 O2a2 South Vietnamese Vietnam
GS000016867 O3c'd'e'f'g'h Southern Han China
GS000019966 O3c'd'e'f'g'h Kankanaey, Luzon Philippines
GS000016986 O3c'd'e'f'g'h Southern Han China
GS000016864 O3c'd'e'f'g'h Southern Han China
GS000016993 O3c'd'e'f'g'h Southern Han China
GS000035253 O3i Batak, Palawan Philippines
GRC15491808 O3i Rai'atea SR27 Society Islands
GS000017006 O3i Sama-Bajaw Sulawesi, Indonesia
GS000022100 O3b Burmese Burma
GS000019904 O3a1 Burmese Burma
GS000019905 O3a1 Burmese Burma
GS000016711 O6b Southern Han China
GS000016676 O6b Southern Han China
GS000016712 O6b Southern Han China
GS000016714 O6b Southern Han China
GS000016850 O6a Southern Han China
GS000016706 O6a Southern Han China
GS000016708 O6a Southern Han China
GS000016660 O6a Southern Han China
GS000016843 O6a Southern Han China
GS000016844 O6a Southern Han China
GRC15491809 O6a Rai'atea SR28 Society Islands
GS000016832 O6a Southern Han China
GS000016683 O6a Southern Han China
GS000016853 O6a Southern Han China
60000
50000
40000
30000
20000
10000
Years before present

Supplementary Figure S12. Reduced median STR network using fifteen loci for hg S-M230 and K*-M9 individuals, constructed using data from this and other studies[8,9,14]. No comparative data were available from western Polynesia at sufficient resolution. Node sizes are proportional to the number of individuals and the smallest node represents one person. Three individuals have genomes included in the Y chromosome tree in Supplementary Fig. S11 (GS000016941 from Sulawesi Bajo, GS000016935 from Borneo Lebbo and GRC15491807 from the Societies). All thirty-four K-P79 examples cluster together in the network (shaded region) and are designated S2a-P79 because the GRC15491807 genome forms a clade with the GS000016935 hg S2-P202 genome in the complete Y phylogenetic tree (Supplementary Fig. S11). The two Societies' haplotypes are adjacent to those from Ontong Java (central northern outlier) and a New Zealand Maori (eastern Polynesia).

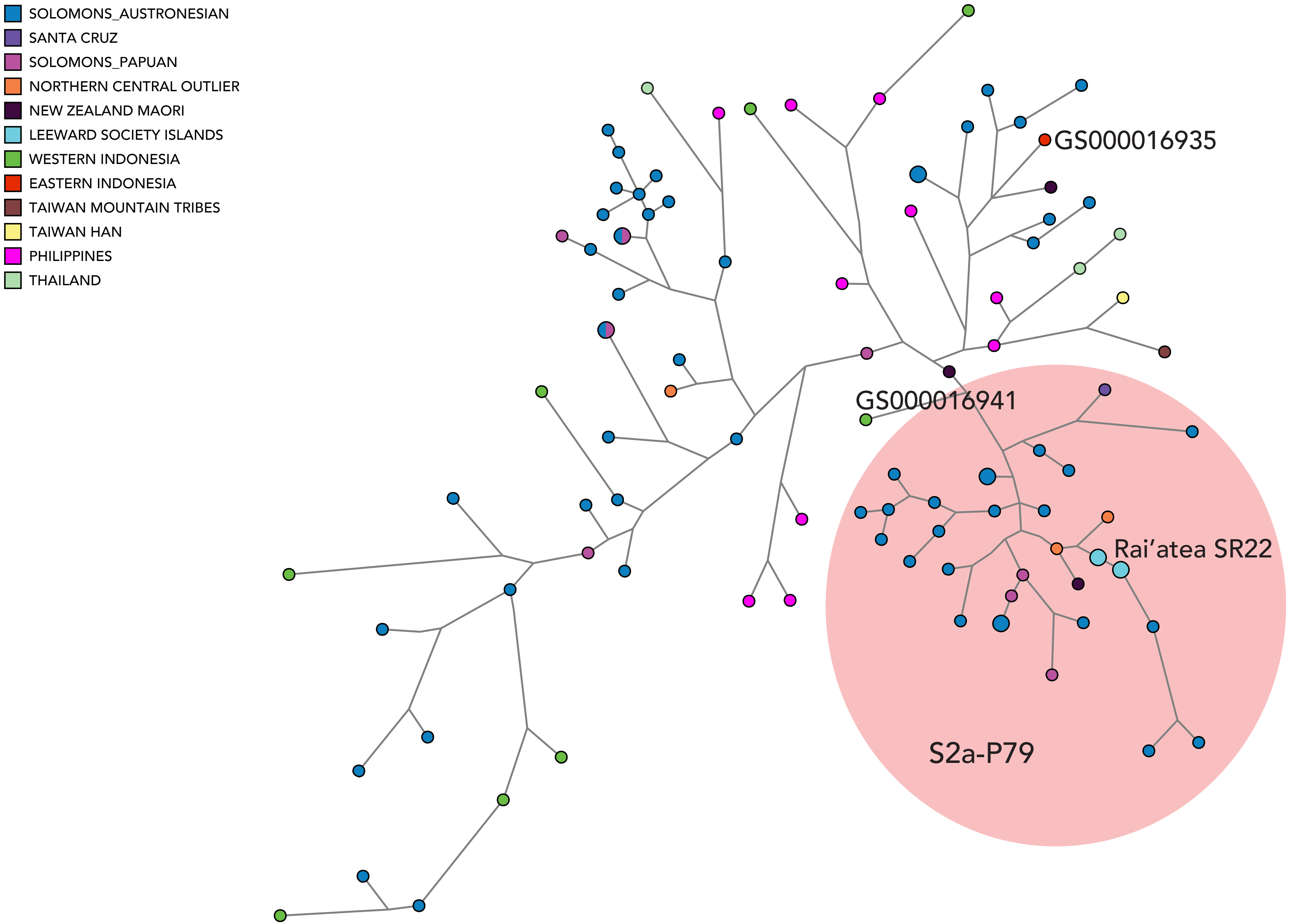
SOLOMONS_AUSTRONESIAN
SANTA CRUZ
SOLOMONS_PAPUAN
NORTHERN CENTRAL OUTLIER
NEW ZEALAND MAORI
LEEWARD SOCIETY ISLANDS
WESTERN INDONESIA
EASTERN INDONESIA
TAIWAN MOUNTAIN TRIBES
TAIWAN HAN
PHILIPPINES
THAILAND
GS000016935
GS000016941
Rai'atea SR22
S2a-P79

References

1 Alexander, D. H., Novembre, J. & Lange, K. Fast model-based estimation of ancestry in unrelated individuals. *Genome research* **19**, 1655-1664 (2009).

2 Hudjashov, G. *et al.* Complex Patterns of Admixture across the Indonesian Archipelago. *Mol Biol Evol* **34**, 2439-2452, doi:10.1093/molbev/msx196 (2017).

3 Patterson, N. *et al.* Ancient admixture in human history. *Genetics* **192**, 1065-1093, doi:10.1534/genetics.112.145037 (2012).

4 Lawson, D. J., Hellenthal, G., Myers, S. & Falush, D. Inference of population structure using dense haplotype data. *PLoS Genet* **8**, e1002453, doi:10.1371/journal.pgen.1002453 (2012).

5 Hellenthal, G. *et al.* A genetic atlas of human admixture history. *Science* **343**, 747-751, doi:10.1126/science.1243518 (2014).

6 R Core Team. *R: A Language and Environment for Statistical Computing*. (R Foundation for Statistical Computing, Vienna, Austria, 2017).

7 Bandelt, H. J., Forster, P., Sykes, B. C. & Richards, M. B. Mitochondrial portraits of human populations using median networks. *Genetics* **141**, 743-753 (1995).

8 Delfin, F. *et al.* Bridging near and remote Oceania: mtDNA and NRY variation in the Solomon Islands. *Mol Biol Evol* **29**, 545-564, doi:10.1093/molbev/msr186 (2012).

9 Karmin, M. *et al.* A recent bottleneck of Y chromosome diversity coincides with a global change in culture. *Genome research* **25**, 459-466, doi:10.1101/gr.186684.114 (2015).

10 Cox, M. P. *et al.* A Polynesian motif on the Y chromosome: population structure in remote Oceania. *Hum Biol* **79**, 525-535, doi:10.1353/hub.2008.0004 (2007).

11 Mirabal, S. *et al.* Increased Y-chromosome resolution of haplogroup O suggests genetic ties between the Ami aborigines of Taiwan and the Polynesian Islands of Samoa and Tonga. *Gene* **492**, 339-348, doi:10.1016/j.gene.2011.10.042 (2012).

12 Drummond, A. J., Suchard, M. A., Xie, D. & Rambaut, A. Bayesian phylogenetics with BEAUti and the BEAST 1.7. *Mol Biol Evol* **29**, 1969-1973, doi:10.1093/molbev/mss075 (2012).

13 Wei, L. H. *et al.* Phylogeography of Y-chromosome haplogroup O3a2b2-N6 reveals patrilineal traces of Austronesian populations on the eastern coastal regions of Asia. *PLoS One* **12**, e0175080, doi:10.1371/journal.pone.0175080 (2017).

14 Trejaut, J. A. *et al*. Taiwan Y-chromosomal DNA variation and its relationship with Island Southeast Asia. *BMC Genet* **15**, 77, doi:10.1186/1471-2156-15-77 (2014).
