# Supplementary material for: Investigating the origins of eastern Polynesians using genome-wide data from the Leeward Society Isles
Source: Sci Rep. 2018 Jan 29;8:1823. doi: 10.1038/s41598-018-20026-8 (PMC5789021; doi:10.1038/s41598-018-20026-8)
Supplement: Supplementary file 2 — Supplementary Table Legends [file 41598_2018_20026_MOESM2_ESM.pdf]

Investigating the origins of eastern Polynesians using genome-wide data from the Leeward Society Isles

Georgi Hudjashov[1,2,8], Phillip Endicott[3,8,*], Helen Post[2], Nano Nagle[4], Simon Y. W. Ho[5], Daniel J. Lawson[6], Maere Reidla[2], Monika Karmin[2], Siiri Rootsi[2], Ene Metspalu[2], Lauri Saag[2], Richard Villems[2], Murray P. Cox[1], R. John Mitchell[4], Ralph L. Garcia-Bertrand[7], Mait Metspalu[2], Rene J. Herrera[7]

[1] Statistics and Bioinformatics Group, Institute of Fundamental Sciences, Massey University, Palmerston North, Manawatu, 4442, New Zealand

[2] Estonian Biocentre, Tartu, Tartumaa, 51010, Estonia

[3] Department Hommes Natures Societies, Musée de l'Homme, 75016, Paris, Ile de France, France

[4] Department of Biochemistry and Genetics, La Trobe University, Melbourne, Victoria, VIC 3086, Australia

[5] School of Life and Environmental Sciences, University of Sydney, Sydney, New South Wales, NSW 2006, Australia

[6] Integrative Epidemiology Unit, School of Social and Community Medicine, University of Bristol, Bristol, BS8 2BN, United Kingdom

[7] Department of Molecular Biology, Colorado College, Colorado Springs, Colorado, 80903, USA

[8] These authors contributed equally to this work

* Correspondence and requests for materials should be addressed to P.E. (email: phillip.endicott@gmail.com)

SUPPLEMENTARY TABLE LEGENDS

Supplementary Table S1. Detailed information about samples used in this study.

Supplementary Table S1A. Summary of new data produced from 81 Leeward Society Islanders and 49 Maori sampled in New Zealand reported in this study.

Supplementary Table S1B. Details of the comparative dataset used for the autosomal analysis. The column '93k SNP dataset' denotes whether an individual was included in the dataset comprised of 93k SNPs and 739 individuals (including data from Hudjashov, et al.[1]), which was used for genotype-based analysis (ADMIXTURE, PCA, *f3* tests and F_{IS}). The column '300k SNP dataset' denotes whether an individual was included in the dataset comprised of 300k SNPs and 570 individuals (excluding data from Hudjashov, et al.[1]), which was used for haplotype-based FS/GT approach and adjunct *f3,* and F_{IS} statistics.

Supplementary Table S2. Details of mtDNA genotyping for 81 individuals from the Leeward Society Isles. Includes details of 25 complete mtDNA genomes generated for this study (shaded in yellow).

Supplementary Table S3. List of results from Y chromosome genotyping for the Leeward Society Isles and Maori sampled in New Zealand.

Supplementary Table S3A. **Details of the hierarchical genotyping of SNPs used to place the Y chromosomes of 81 Leeward Society Islanders and 49 Maori individuals into phylogenetic positions according to the nomenclature of Karmin, et al.[2].** Note that the heterozygous state indicates membership of hg C2a1- P33[3] and this marker was not included in the tree of Karmin, et al.[2] because it lies outside of the non-recombining part of the Y chromosome. Where an individual could not be sub-typed any further, due to difficulties with the assay, or insufficient DNA, it is marked as 'nd' (not determined).

Supplementary Table S3B. **Details of results from microsatellite genotyping conducted on all non-European Leeward Society Y chromosomes (*n*=72) and 49 Maori Y chromosomes**. Missing data is indicated by a blank space. The six positions not tested for in the Maori are denoted by the letters 'NT'.

Supplementary Table S4. List of 541 complete mitochondrial genomes used in the BEAST analyses.

Supplementary Table S4A. Genomes belonging to hg B4a1a (*n*=442).

Supplementary Table S4B. Genomes belonging to hg M29-Q (*n*=111).

Supplementary Table S5. List of 80 Y chromosomes used in the BEAST analysis including the seven produced by target-capture sequencing for this study.

Supplementary Table S6. Coefficient of inbreeding (F_{IS}) estimates in studied populations. Results obtained using three different datasets and sample clustering schemes are shown: a) dataset of *ca* 93k SNPs and 739 samples (with data from Hudjashov, et al.[1]) and the original population affiliation; b) dataset of *ca* 300k SNPs and 570 samples (without data from Hudjashov, et al.[1]) and the original population affiliation; c) as per the approach outlined in (b), but using FS-based population grouping (see Supplementary Table S1B and Materials and Methods for details).

Supplementary Table S7. Results of standard and outgroup *f3* tests[4].

Supplementary Table S7A. Standard *f3* was estimated using the dataset of *ca* 93k SNPs and 739 samples (with data from Hudjashov, et al.[1]) and the original population affiliation. The test was performed using all possible pairwise combinations of non-Polynesian source populations and four Polynesian targets (Leeward Society Islands, Tonga, Samoa, Tahiti).

Supplementary Table S7B. Standard *f3* was estimated using the dataset of *ca* 300k SNPs and 570 samples (without data from Hudjashov, et al.[1]) and the original population affiliation. The test was performed using all possible pairwise combinations of non-Polynesian source populations and Leeward Society Islands as a target.

Supplementary Table S7C. Standard *f3* was estimated using the dataset of *ca* 300k SNPs and 570 samples (without data from Hudjashov, et al.[1]) and FS-based population grouping. The

test was performed using all possible pairwise combinations of non-Polynesian source populations and Leeward Society Islands as a target.

Supplementary Table S7D. Outgroup *f3* was estimated using the dataset of *ca* 93k SNPs and 739 samples (with data from Hudjashov, et al.[1]) and the original population affiliation. African Yoruba (YRI) was used as an outgroup. The test was performed with four Polynesian populations (Leeward Society Islands, Tonga, Samoa, Tahiti).

Supplementary Table S7E. Outgroup *f3* was estimated using the dataset of *ca* 300k SNPs and 570 samples (without data from Hudjashov, et al.[1]) and the original population affiliation. African Yoruba (YRI) was used as an outgroup. The test was performed with Leeward Society Islands only.

Supplementary Table S7F. Outgroup *f3* was estimated using the dataset of *ca* 300k SNPs and 570 samples (without data from Hudjashov, et al.[1]) and FS-based population grouping. African Yoruba (YRI) was used as an outgroup. The test was performed with Leeward Society Islands only.

Supplementary Table S8. Detailed results of the GLOBETROTTER analysis[5]. Results and admixture dates inferred by GT analyses (Figure 3 and Supplementary Fig. S7). The inferred composition of mixing sources for the best-fit events (bar plots on Figure 3 and Supplementary Fig. S7) are shown in the 'sources' section. After accounting for *p*-values, results were considered significant if dates, the CIs of dates, and the composition of mixing sources, overlapped between null.ind=0 and null.ind=1 (as per software guidelines). For an explanation of the null.ind concept and individual summary statistics, refer to Hellenthal, et al.[5] and the GLOBETROTTER manual: https://people.maths.bris.ac.uk/~madjl/finestructure/globetrotter.html.

Supplementary Table S9. Frequencies of mtDNA and Y chromosome haplogroups present in the Leeward Society Isles, and comparative data used from western Polynesia, outlier Polynesia, and Melanesia.

Supplementary Table S9A. **mtDNA haplogroup frequency table.** The table used sources for which complete, or near complete, genomes are available because of the difficulty to

assign individuals to haplogroups using control region sequence data only. The only populations from southern and northern Melanesia displayed are those with matches to the maternal diversity found in Polynesia. All non-zero values are highlighted in red, and regional totals in yellow. Maternal lineages found in the Leeward Societies Isles are shaded in blue and shown in italics.

***Supplementary Table S9B.* Y chromosome haplogroup frequency table.** The table compares all three regions speaking Polynesian languages (western, eastern and outlier Polynesia). The haplogroups are assigned by SNPs and the bi-allelic marker P33. The individuals from Tonga and Samoa were not tested for P33 but according to the median joining network (Supplementary Fig. S9) all but one should be derived at this position. The genotyping of hg O3'7 individuals is also affected by different orders of resolution between data sets, with individuals from the Polynesian outliers not genotyped beyond the O3'6-M324 position[6]. By analysis of microsatellites (not shown) these individuals are inferred to be downstream of the O3a'i-P164 position, rather than the alternative of O6-KL1 (Supplementary Fig. S11). The O6-KL1 ancestral position is used in the table because the individuals from Tutuila (Samoa) were not typed for the downstream position O6a-JST002611 found in the four Leeward Society Individuals. All non-zero values are highlighted in red, and regional totals in yellow. Paternal lineages found in the Leeward Societies Isles are shaded in blue and shown in italics. 'NT' stands for Not Tested.

Supplementary Table S10. List of dates extracted from the BEAST analyses[7].

***Supplementary Table S10A.* List of dates extracted from the BEAST analysis of complete mtDNA sequences for selected nodes of interest with support values greater than 50%.** Median dates for the nodes and the Higher Probability Distributions (HPDs) that exceed the archaeological dates for the Lapita settlement of southern Melanesia and Polynesia are highlighted. The geographic distribution is given.

***Supplementary Table S10B.* List of dates extracted from the BEAST analysis Y chromosome sequences for selected nodes of interest with support values greater than 50%.** Median dates for the nodes and the Higher Probability Distributions (HPDs) that exceed the archaeological dates for the Lapita settlement of southern Melanesia and Polynesia are highlighted.

References

1 Hudjashov, G. *et al*. Complex Patterns of Admixture across the Indonesian Archipelago. *Mol Biol Evol* **34**, 2439-2452, doi:10.1093/molbev/msx196 (2017).

2 Karmin, M. *et al*. A recent bottleneck of Y chromosome diversity coincides with a global change in culture. *Genome research* **25**, 459-466, doi:10.1101/gr.186684.114 (2015).

3 Cox, M. P. *et al*. A Polynesian motif on the Y chromosome: population structure in remote Oceania. *Hum Biol* **79**, 525-535, doi:10.1353/hub.2008.0004 (2007).

4 Patterson, N. *et al*. Ancient admixture in human history. *Genetics* **192**, 1065-1093, doi:10.1534/genetics.112.145037 (2012).

5 Hellenthal, G. *et al*. A genetic atlas of human admixture history. *Science* **343**, 747-751, doi:10.1126/science.1243518 (2014).

6 Delfin, F. *et al*. Bridging near and remote Oceania: mtDNA and NRY variation in the Solomon Islands. *Mol Biol Evol* **29**, 545-564, doi:10.1093/molbev/msr186 (2012).

7 Drummond, A. J., Suchard, M. A., Xie, D. & Rambaut, A. Bayesian phylogenetics with BEAUti and the BEAST 1.7. *Mol Biol Evol* **29**, 1969-1973, doi:10.1093/molbev/mss075 (2012).
